# Fruit and vegetable consumption and its contribution to inequalities in life expectancy and disability-free life expectancy in ten European countries

**DOI:** 10.1007/s00038-019-01253-w

**Published:** 2019-06-11

**Authors:** Adája E. Baars, Jose R. Rubio-Valverde, Yannan Hu, Matthias Bopp, Henrik Brønnum-Hansen, Ramune Kalediene, Mall Leinsalu, Pekka Martikainen, Enrique Regidor, Chris White, Bogdan Wojtyniak, Johan P. Mackenbach, Wilma J. Nusselder

**Affiliations:** 1 Department of Public Health, Erasmus MC, University Medical Center, Dr. Molewaterplein 40, 3015 GD Rotterdam, The Netherlands; 2 Epidemiology, Biostatistics and Prevention Institute, University of Zürich, Zurich, Switzerland; 3 Department of Public Health, University of Copenhagen, Copenhagen, Denmark; 4 Department of Health Management, Lithuanian University of Health Sciences, Kaunas, Lithuania; 5 Stockholm Centre for Health and Social Change, Södertörn University, Stockholm, Sweden; 6 Department of Epidemiology and Biostatistics, National Institute for Health Development, Tallinn, Estonia; 7 Population Research Unit, Faculty of Social Sciences, University of Helsinki, Helsinki, Finland; 8 Department of Public Health and Maternal and Child Health, Faculty of Medicine, Universidad Complutense de Madrid, and CIBER Epidemiología y Salud Pública, Madrid, Spain; 9 Office for National Statistics, Public Policy Analysis Division, London, United Kingdom; 10 Department of Population Health Monitoring and Analysis, National Institute of Public Health, Warsaw, Poland

**Keywords:** Socioeconomic inequalities, Fruit and vegetable consumption, Total life expectancy, Disability-free life expectancy

## Abstract

**Objectives:**

To assess to what extent educational differences in total life expectancy (TLE) and disability-free life expectancy (DFLE) could be reduced by improving fruit and vegetable consumption in ten European countries.

**Methods:**

Data from national census or registries with mortality follow-up, EU-SILC, and ESS were used in two scenarios to calculate the impact: the upward levelling scenario (exposure in low educated equals exposure in high educated) and the elimination scenario (no exposure in both groups). Results are estimated for men and women between ages 35 and 79 years.

**Results:**

Varying by country, upward levelling reduced inequalities in DFLE by 0.1–1.1 years (1–10%) in males, and by 0.0–1.3 years (0–18%) in females. Eliminating exposure reduced inequalities in DFLE between 0.6 and 1.7 years for males (6–15%), and between 0.1 years and 1.8 years for females (3–20%).

**Conclusions:**

Upward levelling of fruit and vegetable consumption would have a small, positive effect on both TLE and DFLE, and could potentially reduce inequalities in TLE and DFLE.

**Electronic supplementary material:**

The online version of this article (10.1007/s00038-019-01253-w) contains supplementary material, which is available to authorized users.

## Introduction

Socioeconomic inequalities in mortality risks are persistent in European countries, although previous research has shown that absolute inequalities between educational groups have decreased among men in several countries in the past decades (de Gelder et al. 2017; Mackenbach et al. 2016). Inequalities in mortality risk between low- and high-educated groups remain an important public health challenge, in particular for preventable causes of death (Mackenbach et al. 2008, 2015b).

In addition to inequalities in mortality, lower educational groups have shorter disability-free life expectancy (DFLE) than higher educational groups. Low socioeconomic groups are, to varying extent when comparing countries, consistently worse off than high socioeconomic groups, with inequalities for DFLE being larger than for total life expectancy (TLE) (Cambois et al. 2016b; Maki et al. 2013). If preventable causes of injury and disease could be reduced in low socioeconomic groups, inequalities between socioeconomic groups in TLE and DFLE would be reduced. Estimating the potential impact of addressing preventable causes and modifiable risk factors allows for both priority setting and for implementation of policies with realistic targets to decrease the inequality between socioeconomic groups.

One modifiable risk factor associated with an increased risk of both mortality and disability is low fruit and vegetable consumption. It has been established as a risk factor for all-cause mortality, with pathways via cardiovascular diseases, cancer, and other, yet unspecified diseases causing increased mortality rates (Agudo et al. 2007; Aune et al. 2017; Bellavia et al. 2013; Genkinger et al. 2004; Leenders et al. 2013; Nguyen et al. 2016; Oyebode et al. 2014; Rissanen et al. 2003). Inverse dose–response relationships for fruit and vegetable consumption and the onset of chronic diseases have been described previously, stressing the risk of consuming inadequate amounts of fruit and vegetables (Bazzano et al. 2002; Dauchet et al. 2006; He et al. 2006; Leenders et al. 2014; Wang et al. 2014a, 2015). Fruit and vegetable consumption varies between educational groups across Europe, with larger differences in Northern European countries than in Mediterranean countries (Prattala et al. 2009). However, a higher level of education is overall associated with a higher consumption of fruit and vegetables (De Irala-Estevez et al. 2000).

This raises the question to what extent educational differences in TLE and DFLE can be reduced by improving fruit and vegetable consumption, similarly as has been shown for mortality rates for smoking (Kulik et al. 2013), obesity (Hoffmann et al. 2015), and alcohol consumption (Mackenbach et al. 2015a). The aim of this study is, therefore, to estimate the impact of improving fruit and vegetable consumption on inequalities in TLE and DFLE between socioeconomic groups in European countries. We evaluate the effect of two scenarios: the upward levelling scenario, where exposure in low educational groups is set to the level of exposure in the high educated, and the elimination scenario, with zero exposure to low fruit and vegetable consumption in each educational group.

## Methods

### Data

Mortality data by age, sex, and level of education were obtained for each country from national census or registries with mortality follow-up including at least data on years 2010 or later, where available. Where no follow-up data were available, we used cross-sectional data provided by the respective countries (see Table 1). Data for Finland, Denmark, United Kingdom, Belgium, Austria, Switzerland, Spain, Poland, Lithuania, and Estonia were included. We included data for ages 35–79 years, excluding age 80 and over since data on mortality by educational level are less reliable in this category.

**Table 1:** Overview of data sources and characteristics for mortality, disability, and fruit and vegetable consumption for males and females, aged 35–79 years, in ten European countries, 2006–2015

Country		Mortality			Disability	Fruit and vegetable consumption
		Period	Person years	Total deaths	EU-SILC 2010 + 2014	ESS Round 7 2014
					Total responses	Total responses
Finland	Male	2010–2014	5,714,996	59,863	8507	1027
	Female	2010–2014	5,929,988	33,987	8550	1060
Denmark	Male	2010–2014	7,463,362	74,614	4548	779
	Female	2010–2014	7,584,952	53,152	4897	723
United Kingdom	Male	2011–2013	410,098	3326	11,902	1052
	Female	2011–2013	434,954	2646	13,330	1211
Belgium	Male	2006–2011	13,273,266	150,621	8456	896
	Female	2006–2011	13,910,896	97,088	9001	873
Austria	Male	2011–2013	4,514,733	41,339	8115	857
	Female	2011–2013	4,772,901	26,486	9106	938
Switzerland	Male	2010–2014	6,027,938	48,202	4824	766
	Female	2010–2014	6,650,291	33,468	5598	766
Spain	Male	2007–2011	49,873,846	504,735	20,698	991
	Female	2007–2011	53,306,240	278,546	22,452	940
Poland^a^	Male	2010–2012	26,822,064	416,485	19,302	737
	Female	2010–2012	29,918,739	241,684	23,182	878
Lithuania	Male	2011–2014	2,771,254	55,972	7052	919
	Female	2011–2014	3,502,529	33,240	9200	1330
Estonia	Male	2012–2015	1,227,024	19,346	7676	835
	Female	2012–2015	1,524,465	12,104	9086	1216
All countries	Male		118,098,581	1,374,503	101,080	8859
	Female		127,535,955	812,401	114,402	9935

*EU*-*SILC* European Union Statistics on Income and Living Conditions, *ESS* European Social Survey

^a^Cross-sectional data

Data on disability prevalence were obtained from the European Union Statistics on Income and Living Conditions (EU-SILC), years 2010 and 2014, for each selected country. These particular years were selected to avoid bias of including respondents multiple times, since EU-SILC is a rotating panel survey. To assess disability, EU-SILC used the Global Activity Limitation Indicator (GALI). It is a validated and relatively accurate indicator, although there are some inconsistencies between countries (Berger et al. 2015; Jagger et al. 2010; Van Oyen et al. 2006, 2018). The GALI consists of one item, asking subjects “For at least the past 6 months, to what extent have you been limited because of a health problem in activities people usually do?” Respondents were classified as having a disability if they responded “Yes, severely” or “Yes, to some extent”. GALI is used to calculate the European disability-free life expectancy indicator “Healthy Life Years” (HLY).

Data on prevalence of low fruit and vegetables by sex, age, educational level, and country were obtained from round 7 (2014) of European Social Survey (ESS). The ESS aims at charting social structure in Europe. Round 7 included a module on health and nutrition (Eikemo et al. 2017). Subjects were asked how many times a day they eat fruit and vegetables in two separate questions. The answering categories were: “Three times or more a day”, “Twice a day”, “Once a day”, “Less than once a day, but at least 4 times a week”, “Less than 4 times a week, but at least once a week”, “Less than once a week”, and “Never”. In our study, fruit and vegetable consumption was considered low if subjects consumed either fruit or vegetables, or both less than once a day. For the countries included in these analyses, response rates range from 43.6 to 68.9%, with high non-response rates observed in the United Kingdom, Austria, Denmark, and Switzerland.

The highest completed level of education was used as an indicator of socioeconomic status. We chose level of education, since it is usually determined early in life, and remains stable during life thereafter. In addition, education was systematically assessed in all three data sources. Level of education was categorized into three levels: low level of education (ISCED 0–2), medium level of education (ISCED 3–4), and high level of education (ISCED 5–6). In the presentation of the results, we focused on inequalities between low level of education and high level of education. Results for medium educated are available in the electronic supplementary material.

We obtained relative risks of low fruit and vegetable consumption on all-cause mortality and disability from the literature. Wang et al. (2014b) reported hazard ratios for mortality attributable to low fruit and vegetable consumption in a meta-analysis, comprising data from seven studies conducted in the United States and Europe, with a total of 553,698 participants, and 42,219 deaths with at least 11 years of follow-up. Estimates of the included studies were adjusted for age, sex, and risk factors such as BMI, alcohol consumption, smoking, and physical activity. We used these hazard ratios to compute a pooled relative risk, weighing them by the size of the corresponding group in ESS (Electronic supplementary material, Table A1). This resulted in a relative risk for mortality in subjects with no daily consumption of fruit and vegetables as compared to those consuming fruit and vegetables at least once a day of 1.2 (95% CI 1.1–1.3).

For disability, fewer studies and no meta-analyses assessing the relationship with fruit and vegetable consumption were available. In our analyses, therefore, we used the relative risk found by Artaud et al. (2013). They assessed the effects of health risk behaviours on several health outcomes, corrected for other risk factors. Their analysis included 3982 French subjects aged 65 and over, a subpopulation of the Three-City Study. Through personal communication, they provided a relative risk that matched the definition of fruit and vegetable consumption in this study. They calculated that the relative risk for this relationship is 1.20 (95% CI 1.06–1.35).

### Statistical methods and models

First, age-standardized mortality rates and prevalences of disability were calculated for each country, using the European Standard population 2013 for descriptive purposes (Eurostat 2013). Restricted cubic spline models were used to smooth weighted age-, gender-, and education-specific prevalences of low fruit and vegetable consumption and prevalence of disability.

Second, population attributable fractions (PAFs) were calculated by combining smoothed prevalences of exposure to a risk factor, specified by age, gender, and level education in the *i*th exposure category (*P*_i_), the prevalence of exposure to a risk factor, specified by age, gender, and level education, in the *i*th exposure category in an alternative exposure scenario ($P_{i}^{\prime }$), and relative risks (RR_*i*_) for the number of exposure categories (*n*) (formula 1).

1 $${\text{PAF}} = \frac{{\mathop \sum \nolimits_{i = 1}^{n} P_{i} {\text{RR}}_{i} - \mathop \sum \nolimits_{i = 1}^{n} P_{i}^{\prime } {\text{RR}}_{i} }}{{\mathop \sum \nolimits_{i = 1}^{n} P_{i} {\text{RR}}_{i} }}$$

Using PAFs, the impact of low fruit and vegetable consumption on mortality rates and disability prevalence in each country was calculated (Hoffmann et al. 2013) for each scenario of exposure (observed, upward levelling, and elimination) by age, gender, and education, as previously explained by Hoffmann et al. (2015). Third, the Sullivan method, an extension of the standard life table method, was used to calculate DFLE (Sullivan 1971). In the Sullivan method, person years are split into years with and without disability by using the prevalence of disability. We used partial TLE and DFLE, which refers to the number of years lived (TLE) or lived free from disability (DFLE) between the ages of 35 and 79. Confidence intervals for these estimates were derived from 1000 bootstrapped samples, taking into account uncertainty for the GALI estimates, fruit and vegetable consumption, and mortality. Uncertainty with regard to the used RRs of low fruit and vegetable consumption on outcomes was not accounted for these samples. Therefore, a sensitivity analysis was conducted, evaluating the impact of imputing alternative values for the relative risks in the PAF calculations.

### Scenarios

Two counterfactual scenarios were carried out. First, an upward levelling scenario, similar to Hoffmann et al. (2015), was calculated, assessing the effect of altering the prevalence of low fruit and vegetable consumption in the low-educated group to the level of the high-educated group. By comparing the result of this scenario to the current situation, the gain that could be achieved in low educated was calculated.

Second, the effect of eliminating exposure to low fruit and vegetable consumption was calculated, by setting the prevalence of low fruit and vegetable consumption to zero in all educational groups. By comparing the result of this elimination scenario to the current situation, the loss in TLE and DFLE due to low fruit and vegetable consumption, or the maximum achievable gain due to zero exposure to low fruit and vegetable consumption was calculated.

## Results

### Prevalence of low fruit and vegetable consumption

Age-standardized prevalences of low fruit and vegetable consumption are presented in Table 2 for each country, stratified by sex, and level of education. In most countries, the prevalence of low fruit and vegetable consumption was highest in the low-educated group. Prevalences were similar in both educational groups in Austrian and Polish males, and in Swiss females. In some populations, exposure to low fruit and vegetables consumption was the lowest in the medium educated (Electronic supplementary material, Table A4). The highest prevalence of low fruit and vegetable consumption was seen for Lithuania. The largest difference between low and high educated was seen in Lithuania as well, where the prevalence of low fruit and vegetable consumption is 40.6% points (males), and 40.2% points (females) higher in the low educated.

**Table 2:** Age-standardized prevalence of low fruit and vegetable consumption with 95% confidence interval for low- and high-educated males and females, aged 35–79 years, for ten European countries, based on the European Social Survey Round 7 (2014)

		Low educated		High educated		Prevalence rate difference (PRD)		Prevalence rate ratio (PRR)	
		Prevalence	95% CI	Prevalence	95% CI	PRD	95% CI	PRR	95% CI
Finland	Male	0.52	0.44 to 0.60	0.37	0.30 to 0.44	0.15	0.03 to 0.29	1.42	1.08 to 1.89
	Female	0.39	0.34 to 0.44	0.18	0.13 to 0.24	0.21	0.06 to 0.34	2.15	1.21 to 3.16
Denmark	Male	0.62	0.57 to 0.68	0.36	0.27 to 0.45	0.26	0.11 to 0.37	1.74	1.24 to 2.21
	Female	0.34	0.29 to 0.40	0.17	0.11 to 0.23	0.17	0.10 to 0.27	2.00	1.48 to 2.70
United Kingdom	Male	0.47	0.41 to 0.53	0.25	0.20 to 0.31	0.22	0.14 to 0.32	1.86	1.50 to 2.59
	Female	0.45	0.40 to 0.51	0.17	0.13 to 0.22	0.28	0.21 to 0.40	2.60	2.07 to 4.41
Belgium	Male	0.50	0.45 to 0.56	0.29	0.21 to 0.38	0.21	0.14 to 0.28	1.72	1.43 to 2.09
	Female	0.37	0.32 to 0.43	0.29	0.22 to 0.36	0.09	− 0.01 to 0.18	1.29	0.99 to 1.79
Austria	Male	0.64	0.59 to 0.68	0.65	0.55 to 0.75	− 0.01	− 0.13 to 0.10	0.99	0.83 to 1.17
	Female	0.53	0.48 to 0.57	0.32	0.23 to 0.42	0.20	0.04 to 0.30	1.63	1.09 to 2.21
Switzerland	Male	0.52	0.46 to 0.59	0.38	0.29 to 0.46	0.15	− 0.04 to 0.24	1.39	0.92 to 1.76
	Female	0.23	0.19 to 0.28	0.24	0.15 to 0.33	− 0.01	− 0.11 to 0.10	0.97	0.65 to 1.64
Spain	Male	0.60	0.56 to 0.65	0.48	0.39 to 0.58	0.12	0.05 to 0.22	1.25	1.20 to 1.56
	Female	0.44	0.40 to 0.49	0.27	0.20 to 0.34	0.17	0.10 to 0.24	1.62	1.32 to 2.06
Poland	Male	0.50	0.45 to 0.56	0.50	0.38 to 0.58	0.01	− 0.20 to 0.17	1.01	0.69 to 1.49
	Female	0.42	0.36 to 0.47	0.17	0.10 to 0.25	0.24	0.11 to 0.35	2.39	1.47 to 3.97
Lithuania	Male	0.81	0.75 to 0.87	0.41	0.30 to 0.52	0.40	0.27 to 0.55	1.98	1.48 to 2.80
	Female	0.65	0.57 to 0.74	0.25	0.17 to 0.33	0.40	0.27 to 0.51	2.60	1.90 to 3.91
Estonia	Male	0.62	0.55 to 0.71	0.48	0.39 to 0.56	0.15	0.01 to 0.28	1.30	1.02 to 1.65
	Female	0.51	0.41 to 0.61	0.29	0.23 to 0.35	0.22	0.12 to 0.34	1.77	1.47 to 2.25
All countries	Male	0.55		0.39		0.16		1.41	
	Female	0.42		0.23		0.19		1.82	

The prevalence rate difference (PRD) is the difference in prevalence of fruit and vegetables consumption between low and high educated. The prevalence rate ratio (PRR) is the ratio of prevalence of low fruit and vegetable consumption in low educated to the prevalence of low fruit and vegetable consumption in high educated

### Total life expectancy

**Fig. 1:**
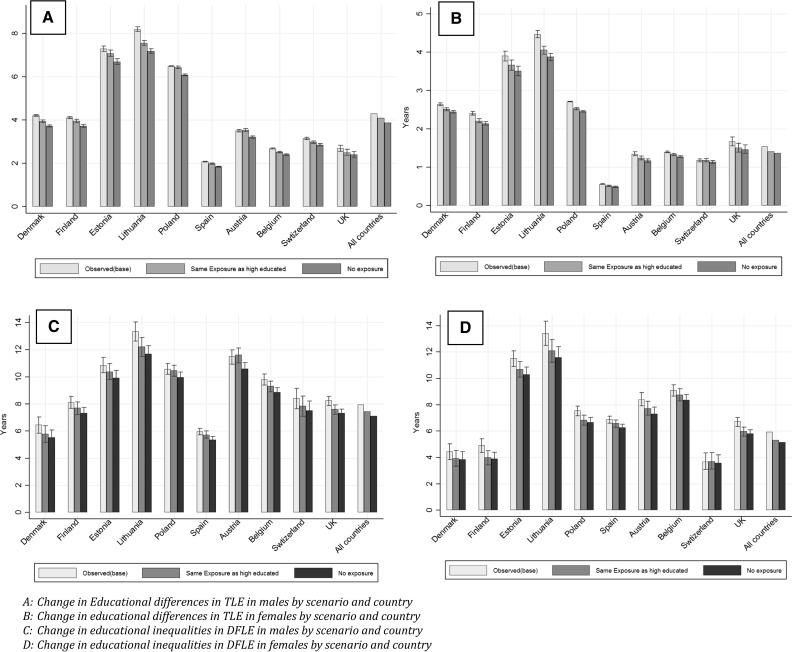
Educational inequalities in total life expectancy, disability-free life expectancy as observed and for the elimination and upward levelling scenarios for men and women between ages 35 and 79 in ten European countries

TLE between the ages of 35 and 80 years varied by country, sex, and educational level (Table 3 and Fig. 1, Electronic supplementary material Table A9). In low educated, the average TLE was 37.2 years for males and 41.2 years for females. In Estonia, Lithuania, and Poland, TLE for low educated was particularly unfavourable compared to other countries, especially for males. The average differences in TLE between low and high-educated groups were 4.3 years for males and 1.5 years for females. The smallest educational differences in TLE between low- and high-educated groups were seen in Spain, with differences of 2.1 years in males and 0.6 years in females. The largest educational differences were seen in Lithuania, with 8.2 years difference in males, and 4.5 years in females.

**Table 3:** Educational differences in total life expectancy and disability-free life expectancy with 95% confidence interval by scenario for males and females, aged 35–79 years, in ten European countries, 2006–2015. The numbers in brackets are confidence intervals

	Observed			Upward Levelling			Elimination			% of total change gap by upward levelling
	Low educated	High educated	Gap low versus high	Gap low versus high	Change gap	Change gap in %	Gap low versus high	Change gap	Change gap in %	
	[A]	[B]	[C]	[D]	[E]	[F]	[G]	[H]	[I]	[J]
**Males**										
Finland										
TLE	37.42 (37.30–37.54)	41.54 (41.48–41.61)	4.11 (3.98–4.26)	3.96 (3.78–4.16)	0.15 (0.01–0.30)	3.73	3.73 (3.57–3.91)	0.38 (0.26–0.51)	9.28	40.3
DFLE	23.65 (23.70–24.63)	31.78 (30.92–32.61)	8.12 (6.85–9.45)	7.70 (6.33–9.04)	0.42 (0.02–0.83)	5.20	7.32 (6.06–8.57)	0.80 (0.43–1.17)	9.89	52.6
Denmark										
TLE	37.49 (37.39–37.59)	41.70 (41.64–41.76)	4.21 (4.10–4.33)	3.94 (3.80–4.10)	0.27 (0.16–0.37)	6.32	3.73 (3.61–3.80)	0.48 (0.41–0.55)	11.49	55.1
DFLE	25.93 (24.58–27.29)	32.40 (31.27–33.51)	6.47 (4.63–8.25)	5.79 (4.00–7.54)	0.67 (0.41–0.95)	10.46	5.53 (3.85–7.17)	0.94 (0.68–1.18)	14.60	71.6
United Kingdom										
TLE	39.40 (39.11–39.71)	42.09 (41.85–42.34)	2.69 (2.31–3.07)	2.50 (2.13–2.88)	0.19 (0.11–0.27)	7.27	2.41 (2.05–2.76)	0.29 (0.22–0.35)	10.84	67.1
DFLE	26.35 (25.64–27.04)	34.60 (33.96–35.18)	8.26 (7.32–9.12)	7.62 (6.64–8.55)	0.64 (0.37–0.88)	7.87	7.33 (6.41–8.21)	0.93 (0.72–1.13)	11.44	68.8
Belgium										
TLE	38.47 (38.42–38.53)	41.16 (41.11–41.21)	2.69 (2.62–2.76)	2.52 (2.41–2.64)	0.16 (0.07–0.25)	6.39	2.41 (2.31–2.50)	0.28 (0.21–0.35)	10.76	59.4
DFLE	24.86 (24.01–25.82)	34.68 (33.97–35.32)	9.81 (8.60–10.85)	9.31 (8.06–10.36)	0.50 (0.21–0.77)	5.39	8.87 (7.69–9.87)	0.95 (0.71–1.17)	10.05	53.7
Austria										
TLE	38.27 (38.13–38.41)	41.77 (41.67–41.86)	3.51 (3.34–3.67)	3.54 (3.35–3.74)	− 0.03 (− 0.14–0.07)	− 0.91	3.21 (3.06–3.38)	0.29 (0.23–0.36)	8.39	− 10.9
DFLE	19.45 (18.11–20.79)	30.95 (30.08–31.76)	11.50 (9.95–13.10)	11.62 (9.99–13.23)	− 0.12 (− 0.50–0.28)	− 1.04	10.60 (9.14–12.03)	0.90 (0.60–1.19)	7.90	− 13.2
Switzerland										
TLE	39.13 (38.99–39.26)	42.29 (42.23–42.35)	3.15 (3.01–3.32)	2.98 (2.81–3.16)	0.17 (0.09–0.26)	5.50	2.85 (2.71–3.01)	0.30 (0.24–0.36)	9.66	57.0
DFLE	25.78 (23.79–28.01)	34.18 (33.34–34.96)	8.41 (6.18–10.59)	7.86 (5.63–9.99)	0.55 (0.28–0.86)	6.70	7.53 (5.43–9.56)	0.88 (0.62–1.16)	10.66	62.8
Spain										
TLE	39.24 (39.22–39.26)	41.32 (41.28–41.35)	2.08 (2.04–2.12)	1.99 (1.91–2.09)	0.08 (0.00–0.16)	4.06	1.85 (1.78–1.92)	0.23 (0.17–0.29)	11.27	36.1
DFLE	28.21 (27.81–28.63)	34.18 (33.55–34.75)	5.97 (5.20–6.67)	5.73 (4.98–6.47)	0.23 (0.01–0.44)	4.02	5.36 (4.66–6.02)	0.61 (0.42–0.78)	10.55	38.1
Poland										
TLE	34.17 (34.16–34.18)	40.66 (40.65–40.67)	6.49 (6.47–6.51)	6.44 (6.37–6.50)	0.05 (− 0.01–0.12)	0.83	6.08 (6.04–6.12)	0.41 (0.38–0.45)	6.33	13.0
DFLE	22.07 (21.78–22.34)	32.64 (32.36–32.95)	10.57 (10.18–10.98)	10.46 (10.03–10.86)	0.11 (− 0.01–0.24)	1.03	9.96 (9.57–10.34)	0.61 (0.52–0.70)	5.68	38.1
Lithuania										
TLE	31.25 (31.02–31.49)	39.43 (39.29–39.58)	8.19 (7.91–8.47)	7.56 (7.21–7.89)	0.63 (0.43–0.83)	7.66	7.18 (6.90–7.48)	1.01 (0.87–1.15)	12.28	62.4
DFLE	19.19 (17.37–21.04)	32.54 (31.60–33.54)	13.36 (11.23–15.52)	12.23 (10.08–14.22)	1.13 (0.75–1.50)	8.46	11.69 (9.72–13.57)	1.66 (1.31–2.04)	12.46	67.9
Estonia										
TLE	32.84 (32.54–33.13)	40.13 (39.93–40.33)	7.29 (6.93–7.65)	7.07 (6.67–7.48)	0.22 (0.02–0.41)	2.97	6.70 (6.32–7.07)	0.59 (0.44–0.74)	8.15	36.4
DFLE	16.65 (15.35–17.86)	27.51 (26.50–28.53)	10.86 (9.26–12.47)	10.38 (8.68–12.01)	0.49 (0.04–0.93)	4.51	9.91 (8.36–11.44)	0.95 (0.52–1.36)	8.62	52.3
All countries										
TLE	37.22	41.50	4.29	4.09	0.19		3.88	0.41		
DFLE	24.82	32.76	7.94	7.46	0.48		7.11	0.83		
**Females**										
Finland										
TLE	40.63 (40.50–40.74)	43.03 (42.98–43.08)	2.40 (2.28–2.54)	2.21 (2.06–2.36)	0.20 (0.11–0.28)	8.00	2.14 (2.00–2.29)	0.26 (0.19–0.33)	10.66	75.1
DFLE	24.33 (23.10–25.50)	29.26 (28.32–30.07)	4.93 (3.43–6.42)	3.99 (2.44–5.50)	0.94 (0.53–1.32)	18.07	3.90 (2.38–5.35)	1.03 (0.65–1.40)	20.02	90.2
Denmark										
TLE	39.95 (39.87–40.04)	42.60 (42.55–42.65)	2.65 (2.54–2.74)	2.51 (2.39–2.64)	0.14 (0.07–0.20)	5.19	2.45 (2.33–2.56)	0.20 (0.14–0.25)	7.58	68.5
DFLE	26.20 (24.98–27.48)	30.64 (29.53–31.78)	4.44 (2.67–6.19)	3.93 (2.12–5.69)	0.51 (0.25–0.74)	11.63	3.85 (2.09–5.58)	0.59 (0.34–0.80)	13.47	86.4
United Kingdom										
TLE	41.04 (40.80–41.28)	42.71 (42.46–42.96)	1.66 (1.33–2.00)	1.50 (1.19–1.83)	0.16 (0.12–0.21)	9.88	1.46 (1.16–1.79)	0.20 (0.16–0.25)	12.21	80.9
DFLE	26.78 (26.14–27.49)	33.52 (33.82–34.20)	6.73 (5.76–7.69)	5.98 (4.98–6.93)	0.75 (0.55–0.96)	7.87	5.81 (4.84–6.73)	0.92 (0.74–1.11)	11.44	81.7
Belgium										
TLE	41.16 (41.11–41.21)	42.56 (42.51–42.61)	1.40 (1.33–1.46)	1.33 (1.25–1.43)	0.07 (0.01–0.12)	4.96	1.28 (1.20–1.36)	0.12 (0.08–0.16)	9.10	54.5
DFLE	24.49 (23.61–25.39)	33.59 (32.69–34.41)	9.11 (7.83–10.28)	8.76 (7.46–9.96)	0.35 (0.06–0.63)	4.07	8.37 (7.13–9.51)	0.74 (0.50–0.96)	8.45	48.2
Austria										
TLE	41.47 (41.39–41.55)	42.82 (42.72–42.93)	1.35 (1.21–1.49)	1.24 (1.09–1.39)	0.11 (0.05–0.17)	8.24	1.17 (1.04–1.31)	0.18 (0.14–0.22)	13.40	61.5
DFLE	22.23 (21.30–23.21)	30.63 (29.55–31.74)	8.41 (6.88–9.94)	7.71 (6.17–9.29)	0.69 (0.31–1.07)	8.45	7.31 (5.86–8.78)	1.10 (0.81–1.37)	13.47	62.8
Switzerland										
TLE	41.94 (41.86–42.02)	43.11 (43.04–43.19)	1.18 (1.06–1.28)	1.18 (1.05–1.30)	0.00 (− 0.05–0.04)	− 0.22	1.14 (1.01–1.24)	0.04 (0.01–0.08)	3.56	− 6.2
DFLE	27.88 (26.55–29.20)	31.57 (30.31–32.74)	3.69 (1.74–5.63)	3.71 (1.78–5.67)	− 0.01 (− 0.31–0.25)	− 0.39	3.58 (1.73–5.43)	0.12 (0.13–0.34)	3.11	− 12.7
Spain										
TLE	42.38 (42.37–42.40)	42.94 (42.91–42.98)	0.56 (0.52–0.60)	0.51 (0.46–0.57)	0.05 (0.01–0.08)	8.38	0.49 (0.44–0.54)	0.07 (0.04–0.10)	13.16	63.6
DFLE	27.91 (27.47–28.34)	34.79 (34.09–35.44)	6.89 (6.05–7.71)	6.59 (5.75–7.44)	0.30 (0.04–0.53)	4.38	6.27 (5.48–7.08)	0.62 (0.43–0.79)	8.99	48.7
Poland										
TLE	39.80 (39.80–39.81)	42.52 (42.51–42.52)	2.71 (2.70–2.72)	2.53 (2.50–2.56)	0.18 (0.15–0.21)	6.64	2.46 (2.43–2.48)	0.25 (0.23–0.27)	9.20	72.1
DFLE	24.75 (24.48–25.03)	32.31 (32.04–32.56)	7.56 (7.16–7.90)	6.85 (6.45–7.22)	0.71 (0.60–0.80)	9.13	6.67 (6.30–7.02)	0.89 (0.80–0.97)	11.55	79.1
Lithuania										
TLE	37.84 (37.58–38.11)	42.32 (42.22–42.40)	4.47 (4.20–4.75)	4.06 (3.75–4.34)	0.42 (0.29–0.53)	9.25	3.88 (3.60–4.13)	0.60 (0.50–0.69)	13.33	69.4
DFLE	21.15 (18.78–23.56)	34.58 (33.64–35.49)	13.43 (10.82–16.10)	12.10 (9.51–14.70)	1.33 (0.92–1.73)	9.87	11.59 (9.14–14.03)	1.84 (1.44–2.24)	13.66	72.2
Estonia										
TLE	38.69 (38.31–39.04)	42.59 (42.48–42.71)	3.90 (3.52–4.27)	3.67 (3.27–4.05)	0.23 (0.10–0.36)	7.01	3.51 (3.13–3.87)	0.39 (0.28–0.50)	10.81	64.8
DFLE	18.04 (16.53–19.58)	29.56 (28.74–30.38)	11.52 (9.88–13.25)	10.69 (8.96–12.45)	0.83 (0.32–1.33)	9.74	10.30 (8.65–11.95)	1.22 (0.77–1.69)	13.03	74.8
All countries										
TLE	41.16	42.70	1.54	1.42	0.13		1.37	0.17		
DFLE	25.72	31.66	5.94	5.32	0.63		5.14	0.80		

Calculations: [C] = [B] − [A]. [E] = [C] − [D]. [H] = [C] − [G]. [F] = [E]/[C]. [I] = [H]/[C]. [J] = [F]/[I]. Columns [D] and [G] can be calculated using Table A9 in the Electronic supplementary material. Since the estimates presented here were rounded after finishing all calculations, reproducing the estimates by hand from this table will yield slightly different results

*TLE* total life expectancy, *DFLE* disability-free life expectancy

In the upward levelling scenario, a reduction in the gap in TLE between low and high educated was seen in almost all countries, with an average reduction of 0.2 years in low-educated males and 0.1 years in low-educated females (Table 3 and Fig. 1, Electronic supplementary material Table A9). Upward levelling had the largest effect in low-educated Lithuanian males with an increase of 0.6 years, and an increase of 0.4 years in women. In other populations, such as Austrian, Polish, and Spanish males, and in Austrian, Belgian, Spanish, and Swiss females, the gains of upward levelling were 0.1 years of TLE or less.

In the elimination scenario, TLE would increase to varying extent in all countries, with larger increases for low-educated groups than for high-educated groups (Table 3 and Fig. 1, Electronic supplementary material Table A9). On average, TLE would increase by 0.6 years from 37.2 years to 37.8 years in low-educated males and 0.2 years in low-educated females, as opposed to increases of 0.2 years in high-educated males and 0.1 years in high-educated females. Possible gains in TLE in low-educated males varied between 0.4 years (UK) and 1.4 years (Lithuania); in low-educated females, possible gains in TLE varied between 0.1 years (Switzerland) and 0.8 years (Lithuania). Inequalities in TLE between educational groups could be reduced by on average 0.4 years in males, ranging from 0.3 years (UK) to 1.0 years (Lithuania), and 0.2 years in females, ranging from 0.0 years (Switzerland) to 0.6 years (Lithuania) for females.

### Disability-free life expectancy

The difference in DFLE between low and high educated was larger than for TLE, with 7.9 years of difference in DFLE for males and 5.9 years for females. Between countries, differences were larger for lower levels of education than for higher levels of education (Table 3 and Fig. 1, Electronic supplementary material A9). DFLE varied between 16.6 years and 28.2 years in low-educated males, and between 18.0 years and 27.9 years in low-educated females. Educational differences in DFLE in Estonia and Lithuania were particularly large, for both males and females.

In the upward levelling scenario, inequalities could be reduced by 0.5 years for males and 0.6 years for females. The largest estimated reductions in the gap between educational groups would be seen in Lithuania, with 1.1 years of DFLE in males, and 1.3 years in females. In other populations, such as Polish males and Swiss females, reductions were practically absent.

The gap in DFLE between educational groups could be reduced by 0.8 years for both males and females. In the elimination scenario, DFLE would improve by 1.5 years in low-educated males, and 1.2 years in low-educated females, and by 0.7 years in high-educated males and 0.4 years in high-educated females. Possible gains in DFLE for low-educated males varied between 1.3 and 2.6 years, and for low-educated females between 0.7 years and 2.4 years (Table 3 and Fig. 1). The gains in DFLE for high-educated individuals varied between 0.4 years (UK) and 1.5 years (Austria) for males, and between 0.3 years (UK) and 0.9 years (Estonia) for females. For males, the possible reduction in the gap in DFLE between educational groups ranged from 0.6 years (Poland, Spain) to 1.7 years (Lithuania). For females, the possible reduction in the gap in DFLE between educational groups ranged from 0.1 years (Switzerland) to 1.8 years (Lithuania).

## Discussion

Improving consumption of fruit and vegetables in low-educated groups to the level of high educated would have a small, but positive effect on both total life expectancy (TLE) and disability-free life expectancy (DFLE), and has the potential to reduce inequalities in health, in particular in countries where inequalities in TLE, DFLE, and fruit and vegetable consumption are large. Zero exposure to low fruit and vegetable consumption would improve TLE and DFLE and decrease educational inequalities in TLE and DFLE, but the effect varies between countries. In more than half of the assessed countries, 50% or more of the potential effect of eliminating low fruit and vegetable consumption could be achieved by upward levelling.

### Strengths and limitations

#### Data

The main advantage of the PAF method is that the best available data of separate sources can be combined into one effect estimate. Longitudinal health surveys generally lack power to assess associations between fruit and vegetable consumption and mortality and disability directly, and providing results for several countries is often difficult.

Consumption of fruit and vegetables was measured in ESS as frequency of use, which introduces uncertainty on total consumption measured in grams. However, previous research indicated that the number of servings of fruit and vegetables correlates with an average consumed amount measured in grams (Nothlings et al. 2006).

Due to cross-sectional assessment of fruit and vegetables consumption in ESS, no statements can be made with regard to duration of exposure. We assumed reported frequencies of consumption to be representative for consumption patterns of a respondent averaged over a longer period of time. However, there are indications that traditional Mediterranean countries, known for their high consumption of fruit and vegetables, and other European countries have grown to be more alike in their consumption patterns than in years past (CIHEAM/FAO 2015). This underlines the difficulty to assess the impact of exposure to low fruit and vegetable consumption, which may vary over time for each individual respondent.

We also compared prevalences of low fruit and vegetable consumption in ESS with data from other sources, namely the DAFNE project, the European Food Safety Authority (EFSA), and the European Health Interview Survey (EHIS) (results not shown). No clear pattern in fruit and vegetable consumption per country could be established when comparing these sources, possibly due to differences in measurement units and sampling design. However, even for data sources using similar measurement units, no clear pattern could be established.

Mortality data obtained from mortality follow-up were supplied per country in a standard format. This improved comparability and allowed for stratification by educational level, sex, and age group. We used cross-sectional data for Poland since no longitudinal data were available, which might introduce selection bias and warrants caution in interpreting the results.

Data on disability were assessed in a similar manner in international surveys. Nonetheless, cultural differences between countries, discrepancies in translations of the questions, and differences between socioeconomic groups in the reporting of disabilities are important issues and should warrant careful interpretation of results (Cambois et al. 2016a). The same may apply to the reporting of fruit and vegetable consumption. Additionally, both data on disability and fruit and vegetable consumption are self-reported, which could lead to both over- and underestimation of disability prevalence and exposure to low fruit and vegetable consumption.

#### Relative risks

For the PAF method, relative risks for mortality and disability in relation to low fruit and vegetable consumption were obtained from the literature. Since no significantly different relative risks specified by country, educational group, or age group were reported, we assumed the effect of fruit and vegetable consumption on all-cause mortality and disability to be the same across countries, educational groups, age groups, and sexes (Artaud et al. 2013; Wang et al. 2014b). A sensitivity analysis by Wang et al. found no significant difference for sex. For disability, we used a RR based on a cohort study among persons aged 65 and over, which might have yielded conservative estimates, as relative risks generally decrease with increase in age.

We conducted a sensitivity analysis to assess the impact of uncertainty around the used relative risks of disability, and, to a lesser extent, all-cause mortality associated with low fruit and vegetable consumption (see  Electronic supplementary material, Table A7). We evaluated several combinations of relative risks. In the first series, we changed the relative risks for mortality and disability from the original values of 1.2 to 1.05 and 1.35 for both mortality and disability. These relative risks are based on the confidence interval for the relative risk reported by Wang et al. (2014b). The effects of upward levelling, calculated in the main analysis, would minimize if the relative risks used in the calculations would decrease, although there might still be a noteworthy effect in Lithuania. In a second series, we kept the relative risk for mortality set at 1.2, while varying the relative risk for disability by 1.02, 1.05, 1.2, and 1.35. The gap in DFLE between low and high educated could potentially be reduced by up to 2.0 years by upward levelling if the relative risk was to be larger. There might be potential for reducing inequalities in DFLE if the relative risk were to be smaller than the relative risk used in the main analysis, although these effects might prove to be not statistically significant.

### Interpretation and comparison with other studies

Our results show that improving consumption of fruit and vegetable consumption in low-educated groups to the level of high-educated groups would have a small, yet positive, effect on both TLE and DFLE in most countries and indicates a potential to reduce inequalities in TLE and DFLE. This was in particular seen in countries where both inequalities in TLE, DFLE, and the differences in prevalence of low fruit and vegetable consumption between low and high educated were large, such as Lithuania. This gradient in fruit and vegetable consumption by level of education in Lithuania has also been described by Kriaucioniene et al. (2012). In the upward levelling scenario, high educated can be regarded as forerunners, and their level of consumption could be viewed as achievable for the entire population of that country.

Since our definition of adequate fruit and vegetable consumption is relatively lenient, improvements for those not meeting this level of consumption are within reach. Additionally, beneficial health effects could be expected if consumption would meet the World Health Organizations recommendation of at least 400 grams of fruits and vegetables a day, since a dose–response relationship for health benefits of fruit and vegetable consumption has been described as well (Wang et al. 2014b; Wiseman 2008). This is in particular the case for countries in Eastern Europe, where the average consumption of fruit and vegetables is further below this WHO recommendation than other European countries (Lock et al. 2005).

A review by McGill has shown that evidence supporting health education interventions was inconclusive, and might even widen socioeconomic inequalities (McGill et al. 2015). However, reducing financial barriers for consuming fruit and vegetables, for example by lowering prices, could be an effective measure to reduce socioeconomic inequalities (McGill et al. 2015). However, further research on successful implementation and the effectiveness of health interventions is necessary.

Our study was the first to assess the impact of fruit and vegetable consumption on educational differences in TLE and DFLE. In the Global Burden of Disease (GBD) study, the impact of a diet low in fruits and a diet low in vegetables on the years of life lost (YLL) and years lived with disability (YLD) was calculated, but not on DFLE nor by level of education. For the total population, we compared their results for mortality, and the percentage of life expectancy with disability (the difference between TLE and DFLE) attributable to low fruit and vegetable consumption to our PAFs (Electronic supplementary material, Table A8). For mortality, results in the GBD study were similar to what we found. For disability, however, we found the fractions in the GBD study to be 3 to 8 times lower than our fractions. These differences for disability may reflect differences in methods and outcome measure, in addition to differences in defining low fruit and vegetable consumption. In the GBD study, only associations between a diet low in fruit or vegetables and the incidence of several diseases, such as cardiovascular disease, type 2 diabetes and neoplasms were included in the calculations. There are indications that low fruit and vegetable consumption is also associated with additional diseases known for causing disability (Boeing et al. 2012), such as cataract (Huang et al. 2015), depression (Liu et al. 2016), and osteoporosis (Luo et al. 2016).

### Conclusion and implications

Improving consumption of fruit and vegetables in low-educated groups to the level of high educated would have a small positive effect on both TLE and DFLE. In particular, in countries where inequalities in TLE, DFLE, and fruit and vegetable consumption are large, such as Lithuania, implementing interventions to improve fruit and vegetable consumption among low-educated groups could be worthwhile. Interventions reducing financial barriers for consuming fruit and vegetables should be considered.

## Electronic supplementary material

Below is the link to the electronic supplementary material.

Supplementary material 1 (DOCX 196 kb)
