# Supplementary material for: Fruit and vegetable consumption and its contribution to inequalities in life expectancy and disability-free life expectancy in ten European countries
Source: Int J Public Health. 2019 Jun 11;64(6):861–72. doi: 10.1007/s00038-019-01253-w (PMC6614160; doi:10.1007/s00038-019-01253-w)
Supplement: Supplementary file 1 — Supplementary material 1 (DOCX 196 kb) [file 38_2019_1253_MOESM1_ESM.docx]

International Journal of Public Health

**Fruit and vegetable consumption and its contribution to inequalities in life expectancy and disability-free life expectancy in 10 European countries**

**Electronic Supplementary Material for:**

Fruit and vegetable consumption and its contribution to inequalities in life expectancy and disability-free life expectancy in 10 European countries.

**Table A1** Pooled relative risk for all-cause mortality in relation to fruit and vegetable consumption based on hazard ratios found by Wang et al., weighed by the European Social Survey Round 7

| Relative Risk | Hazard ratio (Wang et al. 2014) | Beta | ESS (#cases) | ESS % | Beta weighed by ESS % |
| --- | --- | --- | --- | --- | --- |
| Five times a day | 0,66 | -0,42 | 3081 | 8.36% | -0.0347 |
| Four times a day | 0,69 | -0,37 | 5182 | 14.06% | -0.0522 |
| Three times or more a day | 0,73 | -0,31 | 6478 | 17.58% | -0.0553 |
| Twice a day | 0,81 | -0,21 | 10614 | 28.80% | -0.0607 |
| Once a day | 0,90 | -0,11 | 11493 | 31.19% | -0.0329 |
| Less than once a day but at least 4 times a week | 1,00 |  |  |  |  |
| Less than 4 times a week but at least once a week | 1,00 |  |  |  |  |
| Less than once a week | 1,00 |  |  |  |  |
| Never | 1,00 |  |  |  |  |
| Pooled relative risk | 0,7899 |  | 36848 |  | -0,2358 |
| UPPER LIMIT | **1,12659** |  | 36848 | 100,00% | 0,2358 |
|  |  |  |  |  |  |

| Relative Risk | Hazard ratio (Wang et al. 2014) | Beta | ESS (#cases) | ESS % | Beta weighed by ESS % |
| --- | --- | --- | --- | --- | --- |
| Five times a day | 0,74 | -0,30 | 3081 | 8.36% | -0.02518 |
| Four times a day | 0,76 | -0,27 | 5182 | 14.06% | -0.03859 |
| Three times or more a day | 0,79 | -0,24 | 6478 | 17.58% | -0.04144 |
| Twice a day | 0,85 | -0,16 | 10614 | 28.80% | -0.04681 |
| Once a day | 0,92 | -0,08 | 11493 | 31.19% | -0.02601 |
| Less than once a day but at least 4 times a week | 1,00 |  |  |  |  |
| Less than 4 times a week but at least once a week | 1,00 |  |  |  |  |
| Less than once a week | 1,00 |  |  |  |  |
| Never | 1,00 |  |  |  |  |
| Pooled relative risk | 0,837 |  | 36848 |  | -0,17803 |
| POINT ESTIMATE | **1,195** |  | 36848 | 100,00% | 0,17803 |
|  |  |  |  |  |  |

| Relative Risk | Hazard ratio (Wang et al. 2014) | Beta | ESS (#cases) | ESS % | Beta weighed by ESS % |
| --- | --- | --- | --- | --- | --- |
| Five times a day | 0,82 | -0,20 | 3081 | 8.36% | -0.0166 |
| Four times a day | 0,83 | -0,19 | 5182 | 14.06% | -0.0262 |
| Three times or more a day | 0,86 | -0,15 | 6478 | 17.58% | -0.0265 |
| Twice a day | 0,90 | -0,11 | 10614 | 28.80% | -0.0303 |
| Once a day | 0,95 | -0,05 | 11493 | 31.19% | -0.0160 |
| Less than once a day but at least 4 times a week | 1,00 |  |  |  |  |
| Less than 4 times a week but at least once a week | 1,00 |  |  |  |  |
| Less than once a week | 1,00 |  |  |  |  |
| Never | 1,00 |  |  |  |  |
| Pooled relative risk | 0,8908 |  | 36848 |  | -0,1157 |
| LOWER LIMIT | **1,1226** |  | 36848 | 100,00% | 0,1157 |
|  |  |  |  |  |  |

**Table A2** Age standardized mortality rates with 95% confidence interval for males and females, aged 35-79 years, by level of education in 10 European countries, 2006-2015

| Country | Period | ISCED 0-2 | |  |  | ISCED 3-4 |  |  | ISCED 5-6 |  |  |
| --- | --- | --- | --- | --- | --- | --- | --- | --- | --- | --- | --- |
|  |  | **ASMR** | **95% CI** | |  | **ASMR** | **95% CI** |  | **ASMR** | **95% CI** |  |
| Finland |  |  |  | |  |  |  |  |  |  |  |
| *Male* | 2010-2014 | 1180.5 | 1165.5 | | 1196.1 | 901.3 | 888.6 | 914.9 | 559.6 | 548.9 | 570.1 |
| *Female* | 2010-2014 | 621.4 | 608.2 | | 635.3 | 420.4 | 412.7 | 428.9 | 304.8 | 297.1 | 312.6 |
| Denmark |  |  |  | |  |  |  |  |  |  |  |
| *Male* | 2010-2014 | 1204.8 | 1190.5 | | 1219.3 | 840.3 | 831.4 | 849.7 | 555.6 | 545.3 | 565.8 |
| *Female* | 2010-2014 | 781.3 | 770.6 | | 792.3 | 503.0 | 495.4 | 510.7 | 381.7 | 373.3 | 390.3 |
| England/Wales | |  |  | |  |  |  |  |  |  |  |
| *Male* | 2011-2013 | 914.9 | 869.9 | | 958.0 | 565.4 | 508.4 | 623.9 | 467.3 | 428.4 | 507.4 |
| *Female* | 2011-2013 | 613.9 | 582.0 | | 645.1 | 384.8 | 343.8 | 427.0 | 355.9 | 319.9 | 391.9 |
| Belgium |  |  |  | |  |  |  |  |  |  |  |
| *Male* | 2006-2011 | 1052.3 | 1044.8 | | 1060.7 | 828.2 | 817.9 | 839.0 | 616.0 | 607.4 | 624.8 |
| *Female* | 2006-2011 | 566.6 | 561.0 | | 572.2 | 458.1 | 450.8 | 465.6 | 365.3 | 357.6 | 372.5 |
| Austria |  |  |  | |  |  |  |  |  |  |  |
| *Male* | 2011-2013 | 1099.7 | 1077.6 | | 1120.0 | 836.6 | 826.3 | 847.0 | 521.2 | 506.1 | 536.9 |
| *Female* | 2011-2013 | 531.5 | 521.2 | | 541.9 | 423.6 | 415.7 | 431.8 | 338.4 | 319.9 | 357.0 |
| Switzerland | |  |  | |  |  |  |  |  |  |  |
| *Male* | 2010-2014 | 939.1 | 919.3 | | 958.8 | 631.6 | 623.5 | 639.4 | 433.4 | 424.7 | 441.5 |
| *Female* | 2010-2014 | 451.7 | 442.4 | | 461.2 | 323.8 | 318.3 | 329.2 | 290.9 | 279.5 | 302.3 |
| Spain | |  |  | |  |  |  |  |  |  |  |
| *Male* | 2007-2011 | 917.8 | 914.8 | | 920.8 | 748.3 | 742.0 | 755.5 | 613.8 | 607.7 | 620.4 |
| *Female* | 2007-2011 | 395.2 | 393.5 | | 397.0 | 335.7 | 330.6 | 340.9 | 310.8 | 305.1 | 316.4 |
| Poland |  |  |  | |  |  |  |  |  |  |  |
| *Male* | 2010-2012 | 1899.4 | 1892.6 | | 1906.5 | 1114.2 | 1106.5 | 1121.9 | 709.8 | 701.4 | 718.4 |
| *Female* | 2010-2012 | 811.2 | 807.1 | | 815.4 | 536.2 | 531.9 | 540.4 | 534.0 | 504.0 | 565.4 |
| Lithuania | |  |  | |  |  |  |  |  |  |  |
| *Male* | 2011-2014 | 2452.1 | 2405.1 | | 2496.3 | 1644.2 | 1623.8 | 1666.4 | 924.1 | 899.3 | 949.6 |
| *Female* | 2011-2014 | 1058.8 | 1024.3 | | 1095.0 | 612.4 | 602.0 | 622.5 | 386.2 | 379.7 | 392.5 |
| Estonia | |  |  | |  |  |  |  |  |  |  |
| *Male* | 2012-2015 | 2166.4 | 2106.1 | | 2225.5 | 1416.3 | 1387.7 | 1446.4 | 815.3 | 783.9 | 847.7 |
| *Female* | 2012-2015 | 938.8 | 893.5 | | 985.8 | 542.0 | 527.5 | 555.9 | 379.8 | 362.6 | 398.0 |

**Table A3** Age standardized disability prevalences with 95% confidence interval for males and females, aged 35-79 years, by level of education in 10 European countries, 2006-2015

*ASMR* Age standardized mortality rates, *ISCED* International standard classification of education

| Country | | Period | ISCED 0-2 |  |  | ISCED 3-4 |  |  | ISCED 5-6 |  |  |
| --- | --- | --- | --- | --- | --- | --- | --- | --- | --- | --- | --- |
|  | |  | **ASDP** | **95% CI** |  | **ASDP** | **95% CI** |  | **ASDP** | **95% CI** |  |
| Finland | |  |  |  |  |  |  |  |  |  |  |
| *Male* | | 2010-2014 | 0.352 | 0.335 | 0.370 | 0.319 | 0.307 | 0.331 | 0.222 | 0.210 | 0.233 |
| *Female* | | 2010-2014 | 0.391 | 0.366 | 0.415 | 0.363 | 0.351 | 0.375 | 0.298 | 0.286 | 0.310 |
| Denmark | |  |  |  |  |  |  |  |  |  |  |
| *Male* | | 2010-2014 | 0.305 | 0.283 | 0.326 | 0.255 | 0.241 | 0.268 | 0.216 | 0.198 | 0.233 |
| *Female* | | 2010-2014 | 0.384 | 0.359 | 0.409 | 0.302 | 0.287 | 0.317 | 0.277 | 0.260 | 0.294 |
| England/Wales | | |  |  |  |  |  |  |  |  |  |
| *Male* | | 2011-2013 | 0.316 | 0.300 | 0.332 | 0.216 | 0.201 | 0.230 | 0.157 | 0.145 | 0.169 |
| *Female* | | 2011-2013 | 0.326 | 0.310 | 0.342 | 0.228 | 0.214 | 0.242 | 0.190 | 0.177 | 0.203 |
| Belgium |  | |  |  |  |  |  |  |  |  |  |
| *Male* | 2006-2011 | | 0.342 | 0.323 | 0.360 | 0.202 | 0.187 | 0.218 | 0.141 | 0.127 | 0.154 |
| *Female* | 2006-2011 | | 0.305 | 0.283 | 0.326 | 0.264 | 0.248 | 0.280 | 0.187 | 0.171 | 0.203 |
| Austria | | |  |  |  |  |  |  |  |  |  |
| *Male* | 2011-2013 | | 0.469 | 0.441 | 0.498 | 0.324 | 0.310 | 0.337 | 0.240 | 0.222 | 0.257 |
| *Female* | 2011-2013 | | 0.437 | 0.416 | 0.459 | 0.313 | 0.300 | 0.327 | 0.256 | 0.232 | 0.280 |
| Switzerland | | |  |  |  |  |  |  |  |  |  |
| *Male* | | 2010-2014 | 0.325 | 0.289 | 0.361 | 0.230 | 0.214 | 0.246 | 0.175 | 0.159 | 0.192 |
| *Female* | | 2010-2014 | 0.323 | 0.295 | 0.351 | 0.266 | 0.250 | 0.281 | 0.263 | 0.234 | 0.292 |
| Spain | | |  |  |  |  |  |  |  |  |  |
| *Male* | | 2007-2011 | 0.260 | 0.252 | 0.268 | 0.201 | 0.186 | 0.216 | 0.163 | 0.151 | 0.174 |
| *Female* | | 2007-2011 | 0.308 | 0.300 | 0.317 | 0.210 | 0.195 | 0.225 | 0.166 | 0.153 | 0.180 |
| Poland | |  |  |  |  |  |  |  |  |  |  |
| *Male* | | 2010-2012 | 0.352 | 0.334 | 0.370 | 0.262 | 0.254 | 0.270 | 0.175 | 0.160 | 0.189 |
| *Female* | | 2010-2012 | 0.349 | 0.332 | 0.367 | 0.272 | 0.265 | 0.279 | 0.206 | 0.193 | 0.220 |
| Lithuania | | |  |  |  |  |  |  |  |  |  |
| *Male* | | 2011-2014 | 0.349 | 0.321 | 0.377 | 0.255 | 0.241 | 0.268 | 0.161 | 0.143 | 0.180 |
| *Female* | | 2011-2014 | 0.416 | 0.375 | 0.457 | 0.285 | 0.273 | 0.296 | 0.155 | 0.141 | 0.170 |
| Estonia | |  |  |  |  |  |  |  |  |  |  |
| *Male* | | 2012-2015 | 0.480 | 0.452 | 0.508 | 0.359 | 0.345 | 0.374 | 0.284 | 0.266 | 0.303 |
| *Female* | | 2012-2015 | 0.494 | 0.460 | 0.529 | 0.379 | 0.366 | 0.392 | 0.267 | 0.254 | 0.280 |

*ASDP* Age standardized disability prevalences, *ISCED* International standard classification of education

**Table A4** Population attributable fractions for low fruit and vegetable consumption for males and females, aged 35-79 years, by educational group in 10 European countries, 2006-2015

|  | Low educated | Medium educated | High educated |
| --- | --- | --- | --- |
|  | PAF | PAF | PAF |
| Finland |  |  |  |
| *Male* | 0.095 | 0.084 | 0.069 |
| *Female* | 0.072 | 0.058 | 0.035 |
| Denmark |  |  |  |
| *Male* | 0.111 | 0.087 | 0.067 |
| *Female* | 0.064 | 0.063 | 0.033 |
| United Kingdom |  |  |  |
| *Male* | 0.086 | 0.083 | 0.048 |
| *Female* | 0.083 | 0.055 | 0.034 |
| Belgium |  |  |  |
| *Male* | 0.091 | 0.078 | 0.055 |
| *Female* | 0.070 | 0.069 | 0.055 |
| Austria |  |  |  |
| *Male* | 0.113 | 0.094 | 0.115 |
| *Female* | 0.095 | 0.062 | 0.061 |
| Switzerland |  |  |  |
| *Male* | 0.095 | 0.079 | 0.070 |
| *Female* | 0.044 | 0.031 | 0.045 |
| Spain |  |  |  |
| *Male* | 0.108 | 0.069 | 0.088 |
| *Female* | 0.081 | 0.064 | 0.051 |
| Poland |  |  |  |
| *Male* | 0.092 | 0.070 | 0.090 |
| *Female* | 0.077 | 0.053 | 0.034 |
| Lithuania |  |  |  |
| *Male* | 0.139 | 0.098 | 0.076 |
| *Female* | 0.116 | 0.083 | 0.048 |
| Estonia |  |  |  |
| *Male* | 0.111 | 0.093 | 0.087 |
| *Female* | 0.092 | 0.065 | 0.054 |

*PAF* Population attributable fraction.

The population attributable fractions were calculated using age standardized prevalences of fruit and vegetable consumption by educational group.

**Table A5** Age-standardized prevalence of low fruit and vegetable consumption with 95% confidence interval for medium and high educated males and females, aged 35-79 years, in 10 European countries, based on the European Social Survey Round 7 (2014)

|  |  | Medium educated | | High educated | | Prevalence Rate Difference (PRD) | | Prevalence Rate Ratio (PRR) | |
| --- | --- | --- | --- | --- | --- | --- | --- | --- | --- |
|  |  | Prevalence | 95% CI | Prevalence | 95% CI | PRD | 95% CI | PRR | 95% CI |
| Finland | |  |  |  |  |  |  |  |  |
|  | *Male* | 0.46 | 0.40 – 0.51 | 0.37 | 0.30 – 0.44 | 0.15 | 0.03 – 0.29 | 1.42 | 1.08 – 1.89 |
|  | *Female* | 0.31 | 0.26 – 0.36 | 0.18 | 0.13 – 0.24 | 0.21 | 0.06 – 0.34 | 2.15 | 1.21 – 3.16 |
| Denmark | |  |  |  |  |  |  |  |  |
|  | *Male* | 0.48 | 0.35 – 0.60 | 0.36 | 0.27 – 0.45 | 0.26 | 0.11 – 0.37 | 1.74 | 1.24 – 2.21 |
|  | *Female* | 0.34 | 0.26 – 0.41 | 0.17 | 0.11 – 0.23 | 0.17 | 0.10 – 0.27 | 2.00 | 1.48 – 2.70 |
| United Kingdom | |  |  |  |  |  |  |  |  |
|  | *Male* | 0.45 | 0.39 – 0.52 | 0.25 | 0.20 – 0.31 | 0.22 | 0.14 – 0.32 | 1.86 | 1.50 – 2.59 |
|  | *Female* | 0.29 | 0.23 – 0.35 | 0.17 | 0.13 – 0.22 | 0.28 | 0.21 – 0.40 | 2.60 | 2.07 – 4.41 |
| Belgium | |  |  |  |  |  |  |  |  |
| *Male* | | 0.43 | 0.35 – 0.50 | 0.29 | 0.21 – 0.38 | 0.21 | 0.14 – 0.28 | 1.72 | 1.43 – 2.09 |
| *Female* | | 0.38 | 0.30 – 0.45 | 0.29 | 0.22 – 0.36 | 0.09 | -0.01 – 0.18 | 1.29 | 0.99 – 1.79 |
| Austria | |  |  |  |  |  |  |  |  |
|  | *Male* | 0.52 | 0.42 – 0.62 | 0.65 | 0.55 – 0.75 | -0.01 | -0.13 – 0.10 | 0.99 | 0.83 – 1.17 |
|  | *Female* | 0.33 | 0.23 – 0.43 | 0.32 | 0.23 – 0.42 | 0.20 | 0.04 – 0.30 | 1.63 | 1.09 – 2.21 |
| Switzerland | |  |  |  |  |  |  |  |  |
|  | *Male* | 0.43 | 0.35 – 0.51 | 0.38 | 0.29 – 0.46 | 0.15 | -0.04 – 0.24 | 1.39 | 0.92 – 1.76 |
|  | *Female* | 0.16 | 0.10 – 0.22 | 0.24 | 0.15 – 0.33 | -0.01 | -0.11 – 0.10 | 0.97 | 0.65 – 1.64 |
| Spain | |  |  |  |  |  |  |  |  |
|  | *Male* | 0.37 | 0.27 – 0.47 | 0.48 | 0.39 – 0.58 | 0.12 | 0.05 – 0.22 | 1.25 | 1.20 – 1.56 |
|  | *Female* | 0.34 | 0.23 – 0.46 | 0.27 | 0.20 – 0.34 | 0.17 | 0.10 – 0.24 | 1.62 | 1.32 – 2.06 |
| Poland | |  |  |  |  |  |  |  |  |
|  | *Male* | 0.37 | 0.29 – 0.46 | 0.50 | 0.38 – 0.58 | 0.01 | -0.20 – 0.17 | 1.01 | 0.69 – 1.49 |
|  | *Female* | 0.29 | 0.21 – 0.35 | 0.17 | 0.10 – 0.25 | 0.24 | 0.11 – 0.35 | 2.39 | 1.47 – 3.97 |
| Lithuania | |  |  |  |  |  |  |  |  |
|  | *Male* | 0.54 | 0.49 – 0.60 | 0.41 | 0.30 – 0.52 | 0.40 | 0.27 – 0.55 | 1.98 | 1.48 – 2.80 |
|  | *Female* | 0.45 | 0.40 – 0.50 | 0.25 | 0.17 – 0.33 | 0.40 | 0.27 – 0.51 | 2.60 | 1.90 – 3.91 |
| Estonia | |  |  |  |  |  |  |  |  |
|  | *Male* | 0.65 | 0.46 – 0.57 | 0.48 | 0.39 – 0.56 | 0.15 | 0.01 – 0.28 | 1.30 | 1.02 – 1.65 |
|  | *Female* | 0.35 | 0.30 – 0.39 | 0.29 | 0.23 – 0.35 | 0.22 | 0.12 – 0.34 | 1.77 | 1.47 – 2.25 |

The prevalence rate difference (PRD) is the difference in prevalence of fruit and vegetables consumption between low and high educated. The prevalence rate ratio (PRR) is the ratio of prevalence of low fruit and vegetable consumption in low educated to the prevalence of low fruit and vegetable consumption in high educated.

**Table A6** Educational differences in total life expectancy and disability-free life expectancy by scenario for males and females, aged 35-79 years, in 10 European countries, 2006-2015

| MALES | OBSERVED | | | UPWARD LEVELLING | | |  | ELIMINATION | | |  |
| --- | --- | --- | --- | --- | --- | --- | --- | --- | --- | --- | --- |
|  | Medium Educated | High Educated | Gap  med vs high | Medium Educated | High Educated | Gap  med vs high | Change gap (%) | Medium Educated | High Educated | Gap  med vs high | Change gap  (%) |
|  | [A] | [B] | [C] | [D] | [E] | [F] | [G] | [H] | [I] | [J] | [K] |
| Finland |  |  |  |  |  |  |  |  |  |  |  |
| *TLE* | 39.33 | 41.54 | 2.21 | 39.39 | 41.54 | 2.15 | 0.05 | 39.72 | 41.75 | 2.03 | 0.18 |
| *DFLE* | 26.14 | 31.78 | 5.64 | 26.32 | 31.78 | 5.46 | 0.18 | 27.43 | 32.60 | 5.17 | 0.47 |
| Denmark |  |  |  |  |  |  |  |  |  |  |  |
| *TLE* | 40.01 | 41.70 | 1.70 | 40.14 | 41.70 | 1.57 | 0.13 | 40.42 | 41.91 | 1.48 | 0.21 |
| *DFLE* | 29.45 | 32.40 | 2.95 | 29.85 | 32.40 | 2.55 | 0.40 | 30.78 | 33.20 | 2.42 | 0.53 |
| United Kingdom |  |  |  |  |  |  |  |  |  |  |  |
| *TLE* | 41.46 | 42.09 | 0.64 | 41.56 | 42.09 | 0.53 | 0.10 | 41.70 | 42.22 | 0.51 | 0.12 |
| *DFLE* | 31.75 | 34.60 | 2.85 | 32.13 | 34.60 | 2.47 | 0.39 | 32.67 | 35.03 | 2.36 | 0.49 |
| Belgium |  |  |  |  |  |  |  |  |  |  |  |
| *TLE* | 39.83 | 41.16 | 1.33 | 39.91 | 41.16 | 1.26 | 0.07 | 40.15 | 41.35 | 1.20 | 0.13 |
| *DFLE* | 31.08 | 34.68 | 3.59 | 31.28 | 34.68 | 3.39 | 0.20 | 31.95 | 35.17 | 3.22 | 0.37 |
| Austria |  |  |  |  |  |  |  |  |  |  |  |
| *TLE* | 39.84 | 41.77 | 1.93 | 39.74 | 41.77 | 2.03 | -0.10 | 40.27 | 42.11 | 1.84 | 0.09 |
| *DFLE* | 26.04 | 30.95 | 4.91 | 25.68 | 30.95 | 5.27 | -0.36 | 27.63 | 32.43 | 4.81 | 0.10 |
| Switzerland |  |  |  |  |  |  |  |  |  |  |  |
| *TLE* | 41.00 | 42.29 | 1.29 | 41.05 | 42.29 | 1.24 | 0.05 | 41.23 | 42.41 | 1.18 | 0.11 |
| *DFLE* | 30.91 | 34.18 | 3.27 | 31.09 | 34.18 | 3.09 | 0.18 | 31.74 | 34.70 | 2.96 | 0.32 |
| Spain |  |  |  |  |  |  |  |  |  |  |  |
| *TLE* | 40.42 | 41.32 | 0.89 | 40.36 | 41.32 | 0.95 | -0.06 | 40.71 | 41.59 | 0.88 | 0.01 |
| *DFLE* | 31.30 | 34.18 | 2.88 | 31.13 | 34.18 | 3.05 | -0.17 | 32.15 | 34.98 | 2.84 | 0.04 |
| Poland |  |  |  |  |  |  |  |  |  |  |  |
| *TLE* | 38.19 | 40.66 | 2.47 | 38.09 | 40.66 | 2.56 | -0.09 | 38.58 | 40.99 | 2.40 | 0.07 |
| *DFLE* | 27.41 | 32.64 | 5.24 | 27.18 | 32.64 | 5.47 | -0.23 | 28.39 | 33.54 | 5.15 | 0.08 |
| Lithuania |  |  |  |  |  |  |  |  |  |  |  |
| *TLE* | 35.19 | 39.43 | 4.23 | 35.31 | 39.43 | 4.12 | 0.11 | 35.95 | 39.84 | 3.89 | 0.34 |
| *DFLE* | 26.06 | 32.54 | 6.48 | 26.26 | 32.54 | 6.28 | 0.20 | 27.43 | 33.43 | 6.00 | 0.48 |
| Estonia |  |  |  |  |  |  |  |  |  |  |  |
| *TLE* | 36.60 | 40.12 | 3.52 | 36.67 | 40.12 | 3.46 | 0.07 | 37.20 | 40.46 | 3.25 | 0.27 |
| *DFLE* | 22.72 | 27.51 | 4.79 | 22.88 | 27.51 | 4.63 | 0.17 | 24.24 | 28.68 | 4.44 | 0.36 |

| FEMALES | OBSERVED | | | UPWARD LEVELLING | | |  | ELIMINATION | | |  |
| --- | --- | --- | --- | --- | --- | --- | --- | --- | --- | --- | --- |
|  | Medium Educated | High Educated | Gap  med vs high | Medium Educated | High Educated | Gap  med vs high | Change gap (%) | Medium Educated | High Educated | Gap  med vs high | Change gap (%) |
|  | [A] | [B] | [C] | [D] | [E] | [F] | [G] | [H] | [I] | [J] | [K] |
| Finland |  |  |  |  |  |  |  |  |  |  |  |
| *TLE* | 42.21 | 43.03 | 0.82 | 42.26 | 43.03 | 0.77 | 0.05 | 42.33 | 43.08 | 0.75 | 0.07 |
| *DFLE* | 25.80 | 29.26 | 3.46 | 26.15 | 29.26 | 3.12 | 0.34 | 26.66 | 29.69 | 3.03 | 0.42 |
| Denmark |  |  |  |  |  |  |  |  |  |  |  |
| *TLE* | 41.84 | 42.60 | 0.75 | 41.93 | 42.60 | 0.67 | 0.08 | 42.01 | 42.67 | 0.65 | 0.10 |
| *DFLE* | 28.82 | 30.64 | 1.82 | 29.25 | 30.64 | 1.39 | 0.43 | 29.70 | 31.06 | 1.36 | 0.46 |
| United Kingdom |  |  |  |  |  |  |  |  |  |  |  |
| *TLE* | 42.43 | 42.71 | 0.28 | 42.47 | 42.71 | 0.24 | 0.04 | 42.54 | 42.77 | 0.23 | 0.05 |
| *DFLE* | 31.93 | 33.52 | 1.59 | 32.15 | 33.52 | 1.36 | 0.22 | 32.50 | 33.81 | 1.32 | 0.27 |
| Belgium |  |  |  |  |  |  |  |  |  |  |  |
| *TLE* | 41.91 | 42.56 | 0.65 | 41.95 | 42.56 | 0.60 | 0.04 | 42.09 | 42.66 | 0.58 | 0.07 |
| *DFLE* | 29.93 | 33.59 | 3.66 | 30.14 | 33.59 | 3.46 | 0.20 | 30.77 | 34.07 | 3.30 | 0.36 |
| Austria |  |  |  |  |  |  |  |  |  |  |  |
| *TLE* | 42.24 | 42.82 | 0.58 | 42.24 | 42.82 | 0.58 | 0.00 | 42.40 | 42.94 | 0.54 | 0.03 |
| *DFLE* | 27.70 | 30.63 | 2.94 | 27.70 | 30.63 | 2.93 | 0.00 | 28.70 | 31.47 | 2.77 | 0.16 |
| Switzerland |  |  |  |  |  |  |  |  |  |  |  |
| *TLE* | 42.87 | 43.11 | 0.25 | 42.85 | 43.11 | 0.27 | -0.02 | 42.93 | 43.19 | 0.26 | -0.01 |
| *DFLE* | 30.65 | 31.57 | 0.93 | 30.51 | 31.57 | 1.06 | -0.14 | 31.09 | 32.11 | 1.02 | -0.10 |
| Spain |  |  |  |  |  |  |  |  |  |  |  |
| *TLE* | 42.74 | 42.94 | 0.20 | 42.76 | 42.94 | 0.19 | 0.01 | 42.87 | 43.04 | 0.18 | 0.02 |
| *DFLE* | 32.18 | 34.79 | 2.62 | 32.26 | 34.79 | 2.54 | 0.08 | 32.86 | 35.28 | 2.41 | 0.20 |
| Poland |  |  |  |  |  |  |  |  |  |  |  |
| *TLE* | 41.48 | 42.52 | 1.03 | 41.53 | 42.52 | 0.98 | 0.05 | 41.63 | 42.59 | 0.96 | 0.08 |
| *DFLE* | 28.71 | 32.31 | 3.59 | 28.93 | 32.31 | 3.38 | 0.22 | 29.39 | 32.68 | 3.29 | 0.30 |
| Lithuania |  |  |  |  |  |  |  |  |  |  |  |
| *TLE* | 40.91 | 42.31 | 1.41 | 41.04 | 42.31 | 1.28 | 0.13 | 41.24 | 42.46 | 1.22 | 0.19 |
| *DFLE* | 27.64 | 34.58 | 6.94 | 28.17 | 34.58 | 6.41 | 0.54 | 29.04 | 35.14 | 6.09 | 0.85 |
| Estonia |  |  |  |  |  |  |  |  |  |  |  |
| *TLE* | 41.43 | 42.59 | 1.16 | 41.46 | 42.59 | 1.13 | 0.04 | 41.63 | 42.71 | 1.08 | 0.08 |
| *DFLE* | 23.97 | 29.56 | 5.59 | 24.14 | 29.56 | 5.42 | 0.17 | 25.12 | 30.31 | 5.19 | 0.40 |

**Table A7** Sensitivity analysis assessing the impact of uncertainty regarding the relative risks of mortality and disability in relation to low fruit and vegetable consumption on the gap in total life expectancy and disability-free life expectancy between low and high educated men and women in 10 European countries, 2006-2015

| Country | RRm | RRd | Gap TLE obs | Gap TLE UL | change  [1] | Gap  TLE elim | change  [2] | Gap  DFLE obs | Gap  DFLE UL | change  [3] | Gap  DFLE elim | change  [4] | Gap TLE obs | Gap TLE UL | change  [1] | Gap  TLE elim | change  [2] | Gap  DFLE obs | Gap  DFLE UL | change  [3] | Gap  DFLE elim | change  [4] |
| --- | --- | --- | --- | --- | --- | --- | --- | --- | --- | --- | --- | --- | --- | --- | --- | --- | --- | --- | --- | --- | --- | --- |
|  | Men |  |  |  |  |  |  |  |  |  |  |  | Women |  |  |  |  |  |  |  |  |  |
| Finland | 1.05 | 1.05 | 4.11 | 4.07 | -0.04 | 4.01 | -0.10 | 8.12 | 8.01 | -0.11 | 7.91 | -0.22 | 2.40 | 2.35 | -0.05 | 2.33 | -0.07 | 4.93 | 4.68 | -0.25 | 4.65 | -0.28 |
| Finland | 1.20 | 1.02 | 4.11 | 3.96 | -0.15 | 3.73 | -0.38 | 8.12 | 8.00 | -0.12 | 7.88 | -0.25 | 2.40 | 2.21 | -0.20 | 2.14 | -0.26 | 4.93 | 4.74 | -0.19 | 4.70 | -0.23 |
| Finland | 1.20 | 1.05 | 4.11 | 3.96 | -0.15 | 3.73 | -0.38 | 8.12 | 7.95 | -0.17 | 7.77 | -0.35 | 2.40 | 2.21 | -0.20 | 2.14 | -0.26 | 4.93 | 4.60 | -0.32 | 4.55 | -0.38 |
| Finland | **1.20** | **1.20** | **4.11** | **3.96** | **-0.15** | **3.73** | **-0.38** | **8.12** | **7.70** | **-0.42** | **7.32** | **-0.80** | **2.40** | **2.21** | **-0.20** | **2.14** | **-0.26** | **4.93** | **3.99** | **-0.94** | **3.90** | **-1.03** |
| Finland | 1.20 | 1.35 | 4.11 | 3.96 | -0.15 | 3.73 | -0.38 | 8.12 | 7.49 | -0.64 | 6.94 | -1.18 | 2.40 | 2.21 | -0.20 | 2.14 | -0.26 | 4.93 | 3.46 | -1.47 | 3.35 | -1.58 |
| Finland | 1.35 | 1.35 | 4.11 | 3.86 | -0.25 | 3.49 | -0.63 | 8.12 | 7.43 | -0.69 | 6.81 | -1.31 | 2.40 | 2.08 | -0.33 | 1.97 | -0.43 | 4.93 | 3.38 | -1.55 | 3.25 | -1.68 |
|  | Men |  |  |  |  |  |  |  |  |  |  |  | Women |  |  |  |  |  |  |  |  |  |
| Denmark | 1.05 | 1.05 | 4.21 | 4.14 | -0.07 | 4.08 | -0.13 | 6.47 | 6.29 | -0.18 | 6.21 | -0.26 | 2.65 | 2.61 | -0.04 | 2.59 | -0.05 | 4.44 | 4.30 | -0.13 | 4.28 | -0.16 |
| Denmark | 1.20 | 1.02 | 4.21 | 3.94 | -0.27 | 3.73 | -0.48 | 6.47 | 6.24 | -0.23 | 6.09 | -0.37 | 2.65 | 2.51 | -0.14 | 2.45 | -0.20 | 4.44 | 4.31 | -0.13 | 4.27 | -0.17 |
| Denmark | 1.20 | 1.05 | 4.21 | 3.94 | -0.27 | 3.73 | -0.48 | 6.47 | 6.16 | -0.31 | 5.99 | -0.48 | 2.65 | 2.51 | -0.14 | 2.45 | -0.20 | 4.44 | 4.24 | -0.20 | 4.19 | -0.25 |
| Denmark | **1.20** | **1.20** | **4.21** | **3.94** | **-0.27** | **3.73** | **-0.48** | **6.47** | **5.79** | **-0.67** | **5.53** | **-0.94** | **2.65** | **2.51** | **-0.14** | **2.45** | **-0.20** | **4.44** | **3.93** | **-0.51** | **3.85** | **-0.59** |
| Denmark | 1.20 | 1.35 | 4.21 | 3.94 | -0.27 | 3.73 | -0.48 | 6.47 | 5.49 | -0.98 | 5.16 | -1.31 | 2.65 | 2.51 | -0.14 | 2.45 | -0.20 | 4.44 | 3.64 | -0.80 | 3.54 | -0.89 |
| Denmark | 1.35 | 1.35 | 4.21 | 3.78 | -0.43 | 3.43 | -0.78 | 6.47 | 5.37 | -1.10 | 4.95 | -1.52 | 2.65 | 2.42 | -0.23 | 2.31 | -0.33 | 4.44 | 3.58 | -0.86 | 3.46 | -0.98 |
|  | Men |  |  |  |  |  |  |  |  |  |  |  | Women |  |  |  |  |  |  |  |  |  |
| United Kingdom | 1.05 | 1.05 | 2.69 | 2.64 | -0.05 | 2.62 | -0.08 | 8.26 | 8.09 | -0.17 | 8.01 | -0.25 | 1.66 | 1.62 | -0.04 | 1.61 | -0.05 | 6.73 | 6.54 | -0.20 | 6.49 | -0.25 |
| United Kingdom | 1.20 | 1.02 | 2.69 | 2.50 | -0.19 | 2.41 | -0.29 | 8.26 | 8.09 | -0.17 | 8.02 | -0.23 | 1.66 | 1.50 | -0.16 | 1.46 | -0.20 | 6.73 | 6.57 | -0.16 | 6.54 | -0.20 |
| United Kingdom | 1.20 | 1.05 | 2.69 | 2.50 | -0.19 | 2.41 | -0.29 | 8.26 | 8.00 | -0.25 | 7.90 | -0.36 | 1.66 | 1.50 | -0.16 | 1.46 | -0.20 | 6.73 | 6.47 | -0.27 | 6.41 | -0.33 |
| United Kingdom | **1.20** | **1.20** | **2.69** | **2.50** | **-0.19** | **2.41** | **-0.29** | **8.26** | **7.62** | **-0.64** | **7.33** | **-0.93** | **1.66** | **1.50** | **-0.16** | **1.46** | **-0.20** | **6.73** | **5.98** | **-0.75** | **5.81** | **-0.92** |
| United Kingdom | 1.20 | 1.35 | 2.69 | 2.50 | -0.19 | 2.41 | -0.29 | 8.26 | 7.28 | -0.98 | 6.84 | -1.42 | 1.66 | 1.50 | -0.16 | 1.46 | -0.20 | 6.73 | 5.55 | -1.18 | 5.30 | -1.44 |
| United Kingdom | 1.35 | 1.35 | 2.69 | 2.38 | -0.32 | 2.22 | -0.47 | 8.26 | 7.20 | -1.05 | 6.73 | -1.52 | 1.66 | 1.40 | -0.27 | 1.33 | -0.33 | 6.73 | 5.49 | -1.25 | 5.22 | -1.52 |
|  | Men |  |  |  |  |  |  |  |  |  |  |  | Women |  |  |  |  |  |  |  |  |  |
| Belgium | 1.05 | 1.05 | 2.69 | 2.64 | -0.04 | 2.61 | -0.08 | 9.81 | 9.68 | -0.13 | 9.56 | -0.25 | 1.40 | 1.38 | -0.02 | 1.37 | -0.03 | 9.11 | 9.01 | -0.09 | 8.91 | -0.20 |
| Belgium | 1.20 | 1.02 | 2.69 | 2.52 | -0.16 | 2.41 | -0.28 | 9.81 | 9.67 | -0.14 | 9.59 | -0.22 | 1.40 | 1.33 | -0.07 | 1.28 | -0.12 | 9.11 | 9.04 | -0.07 | 9.59 | -0.22 |
| Belgium | 1.20 | 1.05 | 2.69 | 2.52 | -0.16 | 2.41 | -0.28 | 9.81 | 9.61 | -0.20 | 9.46 | -0.35 | 1.40 | 1.33 | -0.07 | 1.28 | -0.12 | 9.11 | 8.99 | -0.12 | 8.87 | -0.24 |
| Belgium | **1.20** | **1.20** | **2.69** | **2.52** | **-0.16** | **2.41** | **-0.28** | **9.81** | **9.31** | **-0.50** | **8.87** | **-0.95** | **1.40** | **1.33** | **-0.07** | **1.28** | **-0.12** | **9.11** | **8.76** | **-0.35** | **8.37** | **-0.74** |
| Belgium | 1.20 | 1.35 | 2.69 | 2.52 | -0.16 | 2.41 | -0.28 | 9.81 | 9.05 | -0.76 | 8.35 | -1.46 | 1.40 | 1.33 | -0.07 | 1.28 | -0.12 | 9.11 | 8.55 | -0.56 | 7.92 | -1.19 |
| Belgium | 1.35 | 1.35 | 2.69 | 2.41 | -0.27 | 2.23 | -0.46 | 9.81 | 8.98 | -0.83 | 8.26 | -1.55 | 1.40 | 1.29 | -0.11 | 1.20 | -0.20 | 9.11 | 8.52 | -0.58 | 7.88 | -1.23 |
|  | Men |  |  |  |  |  |  |  |  |  |  |  | Women |  |  |  |  |  |  |  |  |  |
| Austria | 1.05 | 1.05 | 3.51 | 3.52 | 0.01 | 3.43 | -0.08 | 11.50 | 11.53 | 0.03 | 11.26 | -0.24 | 1.35 | 1.32 | -0.03 | 1.30 | -0.05 | 8.41 | 8.22 | -0.19 | 8.11 | -0.30 |
| Austria | 1.20 | 1.02 | 3.51 | 3.54 | 0.03 | 3.21 | -0.29 | 11.50 | 11.52 | 0.02 | 11.38 | -0.12 | 1.35 | 1.24 | -0.11 | 1.17 | -0.18 | 8.41 | 8.29 | -0.12 | 8.24 | -0.17 |
| Austria | 1.20 | 1.05 | 3.51 | 3.54 | 0.03 | 3.21 | -0.29 | 11.50 | 11.54 | 0.04 | 11.23 | -0.26 | 1.35 | 1.24 | -0.11 | 1.17 | -0.18 | 8.41 | 8.19 | -0.22 | 8.07 | -0.34 |
| Austria | **1.20** | **1.20** | **3.51** | **3.54** | **0.03** | **3.21** | **-0.29** | **11.50** | **11.62** | **0.12** | **10.60** | **-0.90** | **1.35** | **1.24** | **-0.11** | **1.17** | **-0.18** | **8.41** | **7.71** | **-0.69** | **7.31** | **-1.10** |
| Austria | 1.20 | 1.35 | 3.51 | 3.54 | 0.03 | 3.21 | -0.29 | 11.50 | 11.69 | 0.19 | 10.06 | -1.44 | 1.35 | 1.24 | -0.11 | 1.17 | -0.18 | 8.41 | 7.30 | -1.11 | 6.66 | -1.74 |
| Austria | 1.35 | 1.35 | 3.51 | 3.56 | 0.05 | 3.03 | -0.48 | 11.50 | 11.70 | 0.20 | 10.01 | -1.49 | 1.35 | 1.17 | -0.18 | 1.06 | -0.29 | 8.41 | 7.27 | -1.14 | 6.62 | -1.79 |
|  | Men |  |  |  |  |  |  |  |  |  |  |  | Women |  |  |  |  |  |  |  |  |  |
| Switzerland | 1.05 | 1.05 | 3.15 | 3.11 | -0.05 | 3.07 | -0.08 | 8.41 | 8.26 | -0.15 | 8.17 | -0.24 | 1.18 | 1.18 | 0.00 | 1.17 | -0.01 | 3.69 | 3.70 | 0.00 | 3.66 | -0.03 |
| Switzerland | 1.20 | 1.02 | 3.15 | 2.98 | -0.17 | 2.85 | -0.30 | 8.41 | 8.26 | -0.15 | 8.18 | -0.23 | 1.18 | 1.18 | 0.00 | 1.14 | -0.04 | 3.69 | 3.69 | 0.00 | 3.66 | -0.03 |
| Switzerland | 1.20 | 1.05 | 3.15 | 2.98 | -0.17 | 2.85 | -0.30 | 8.41 | 8.19 | -0.22 | 8.06 | -0.35 | 1.18 | 1.18 | 0.00 | 1.14 | -0.04 | 3.69 | 3.70 | 0.00 | 3.65 | -0.04 |
| Switzerland | **1.20** | **1.20** | **3.15** | **2.98** | **-0.17** | **2.85** | **-0.30** | **8.41** | **7.86** | **-0.55** | **7.53** | **-0.88** | **1.18** | **1.18** | **0.00** | **1.14** | **-0.04** | **3.69** | **3.71** | **0.01** | **3.58** | **-0.12** |
| Switzerland | 1.20 | 1.35 | 3.15 | 2.98 | -0.17 | 2.85 | -0.30 | 8.41 | 7.56 | -0.84 | 7.08 | -1.33 | 1.18 | 1.18 | 0.00 | 1.14 | -0.04 | 3.69 | 3.72 | 0.02 | 3.51 | -0.18 |
| Switzerland | 1.35 | 1.35 | 3.15 | 2.87 | -0.28 | 2.66 | -0.49 | 8.41 | 7.50 | -0.91 | 6.97 | -1.44 | 1.18 | 1.18 | 0.00 | 1.11 | -0.07 | 3.69 | 3.72 | 0.02 | 3.49 | -0.20 |
|  | Men |  |  |  |  |  |  |  |  |  |  |  | Women |  |  |  |  |  |  |  |  |  |
| Spain | 1.05 | 1.05 | 2.08 | 2.06 | -0.02 | 2.01 | -0.06 | 5.97 | 5.90 | -0.06 | 5.80 | -0.16 | 0.56 | 0.55 | -0.01 | 0.54 | -0.02 | 6.89 | 6.81 | -0.08 | 6.72 | -0.16 |
| Spain | 1.20 | 1.02 | 2.08 | 1.99 | -0.08 | 1.85 | -0.23 | 5.97 | 5.90 | -0.07 | 5.80 | -0.16 | 0.56 | 0.51 | -0.05 | 0.49 | -0.07 | 6.89 | 6.83 | -0.05 | 6.80 | -0.09 |
| Spain | 1.20 | 1.05 | 2.08 | 1.99 | -0.08 | 1.85 | -0.23 | 5.97 | 5.87 | -0.10 | 5.72 | -0.24 | 0.56 | 0.51 | -0.05 | 0.49 | -0.07 | 6.89 | 6.79 | -0.10 | 6.71 | -0.18 |
| Spain | **1.20** | **1.20** | **2.08** | **1.99** | **-0.08** | **1.85** | **-0.23** | **5.97** | **5.73** | **-0.23** | **5.36** | **-0.61** | **0.56** | **0.51** | **-0.05** | **0.49** | **-0.07** | **6.89** | **6.59** | **-0.30** | **6.27** | **-0.62** |
| Spain | 1.20 | 1.35 | 2.08 | 1.99 | -0.08 | 1.85 | -0.23 | 5.97 | 5.62 | -0.35 | 5.05 | -0.92 | 0.56 | 0.51 | -0.05 | 0.49 | -0.07 | 6.89 | 6.40 | -0.48 | 5.89 | -1.00 |
| Spain | 1.35 | 1.35 | 2.08 | 1.94 | -0.14 | 1.70 | -0.38 | 5.97 | 5.58 | -0.38 | 4.97 | -0.99 | 0.56 | 0.48 | -0.08 | 0.44 | -0.12 | 6.89 | 6.39 | -0.50 | 5.87 | -1.02 |
|  | Men |  |  |  |  |  |  |  |  |  |  |  | Women |  |  |  |  |  |  |  |  |  |
| Poland | 1.05 | 1.05 | 6.49 | 6.48 | -0.01 | 6.38 | -0.11 | 10.57 | 10.54 | -0.03 | 10.41 | -0.16 | 2.71 | 2.66 | -0.05 | 2.64 | -0.07 | 7.56 | 7.37 | -0.19 | 7.32 | -0.24 |
| Poland | 1.20 | 1.02 | 6.49 | 6.44 | -0.05 | 6.08 | -0.41 | 10.57 | 10.54 | -0.04 | 10.36 | -0.22 | 2.71 | 2.53 | -0.18 | 2.46 | -0.25 | 7.56 | 7.41 | -0.15 | 7.36 | -0.20 |
| Poland | 1.20 | 1.05 | 6.49 | 6.44 | -0.05 | 6.08 | -0.41 | 10.57 | 10.52 | -0.05 | 10.28 | -0.29 | 2.71 | 2.53 | -0.18 | 2.46 | -0.25 | 7.56 | 7.31 | -0.25 | 7.24 | -0.32 |
| Poland | **1.20** | **1.20** | **6.49** | **6.44** | **-0.05** | **6.08** | **-0.41** | **10.57** | **10.46** | **-0.11** | **9.96** | **-0.61** | **2.71** | **2.53** | **-0.18** | **2.46** | **-0.25** | **7.56** | **6.85** | **-0.71** | **6.67** | **-0.89** |
| Poland | 1.20 | 1.35 | 6.49 | 6.44 | -0.05 | 6.08 | -0.41 | 10.57 | 10.41 | -0.16 | 9.69 | -0.88 | 2.71 | 2.53 | -0.18 | 2.46 | -0.25 | 7.56 | 6.45 | -1.12 | 6.18 | -1.38 |
| Poland | 1.35 | 1.35 | 6.49 | 6.40 | -0.09 | 5.81 | -0.68 | 10.57 | 10.39 | -0.18 | 9.55 | -1.02 | 2.71 | 2.41 | -0.30 | 2.29 | -0.42 | 7.56 | 6.38 | -1.18 | 6.09 | -1.47 |
|  | Men |  |  |  |  |  |  |  |  |  |  |  | Women |  |  |  |  |  |  |  |  |  |
| Lithuania | 1.05 | 1.05 | 8.21 | 8.04 | -0.17 | 7.93 | -0.27 | 13.37 | 13.05 | -0.31 | 12.91 | -0.45 | 4.50 | 4.38 | -0.11 | 4.33 | -0.16 | 13.44 | 13.07 | -0.36 | 12.93 | -0.50 |
| Lithuania | 1.20 | 1.02 | 8.21 | 7.58 | -0.63 | 7.20 | -1.01 | 13.37 | 12.95 | -0.42 | 12.78 | -0.59 | 4.50 | 4.08 | -0.42 | 3.90 | -0.60 | 13.44 | 13.13 | -0.31 | 13.03 | -0.40 |
| Lithuania | 1.20 | 1.05 | 8.21 | 7.58 | -0.63 | 7.20 | -1.01 | 13.37 | 12.82 | -0.55 | 12.57 | -0.79 | 4.50 | 4.08 | -0.42 | 3.90 | -0.60 | 13.44 | 12.94 | -0.49 | 12.77 | -0.67 |
| Lithuania | **1.20** | **1.20** | **8.21** | **7.58** | **-0.63** | **7.20** | **-1.01** | **13.37** | **12.24** | **-1.13** | **11.70** | **-1.66** | **4.50** | **4.08** | **-0.42** | **3.90** | **-0.60** | **13.44** | **12.11** | **-1.33** | **11.60** | **-1.84** |
| Lithuania | 1.20 | 1.35 | 8.21 | 7.58 | -0.63 | 7.20 | -1.01 | 13.37 | 11.77 | -1.60 | 11.03 | -2.33 | 4.50 | 4.08 | -0.42 | 3.90 | -0.60 | 13.44 | 11.41 | -2.02 | 10.65 | -2.78 |
| Lithuania | 1.35 | 1.35 | 8.21 | 7.20 | -1.01 | 6.58 | -1.63 | 13.37 | 11.56 | -1.81 | 10.68 | -2.69 | 4.50 | 3.82 | -0.67 | 3.53 | -0.96 | 13.44 | 11.29 | -2.15 | 10.47 | -2.96 |
|  | Men |  |  |  |  |  |  |  |  |  |  |  | Women |  |  |  |  |  |  |  |  |  |
| Estonia | 1.05 | 1.05 | 7.30 | 7.24 | -0.06 | 7.14 | -0.16 | 10.87 | 10.73 | -0.13 | 10.61 | -0.25 | 3.91 | 3.85 | -0.06 | 3.81 | -0.10 | 11.53 | 11.30 | -0.22 | 11.20 | -0.33 |
| Estonia | 1.20 | 1.02 | 7.30 | 7.08 | -0.22 | 6.70 | -0.59 | 10.87 | 10.74 | -0.13 | 10.59 | -0.28 | 3.91 | 3.68 | -0.23 | 3.52 | -0.39 | 11.53 | 11.37 | -0.16 | 11.29 | -0.24 |
| Estonia | 1.20 | 1.05 | 7.30 | 7.08 | -0.22 | 6.70 | -0.59 | 10.87 | 10.67 | -0.19 | 10.46 | -0.40 | 3.91 | 3.68 | -0.23 | 3.52 | -0.39 | 11.53 | 11.24 | -0.28 | 11.11 | -0.41 |
| Estonia | **1.20** | **1.20** | **7.30** | **7.08** | **-0.22** | **6.70** | **-0.59** | **10.87** | **10.38** | **-0.49** | **9.91** | **-0.95** | **3.91** | **3.68** | **-0.23** | **3.52** | **-0.39** | **11.53** | **10.70** | **-0.83** | **10.31** | **-1.22** |
| Estonia | 1.20 | 1.35 | 7.30 | 7.08 | -0.22 | 6.70 | -0.59 | 10.87 | 10.13 | -0.74 | 9.47 | -1.40 | 3.91 | 3.68 | -0.23 | 3.52 | -0.39 | 11.53 | 10.23 | -1.30 | 9.63 | -1.90 |
| Estonia | 1.35 | 1.35 | 7.30 | 6.94 | -0.35 | 6.32 | -0.98 | 10.87 | 10.07 | -0.80 | 9.30 | -1.56 | 3.91 | 3.53 | -0.38 | 3.28 | -0.64 | 11.53 | 10.17 | -1.36 | 9.53 | -2.00 |

*RRm* relative risk of mortality in relation to low fruit and vegetable consumption, *RRd* relative risk of disability in relation to low fruit and vegetable consumption, *Gap TLE obs* gap in total life expectancy between low and high educated, as observed in the data, *Gap TLE UL* gap in total life expectancy between low and high educated for the upward levelling scenario, *Gap TLE elim* gap in total life expectancy between low and high educated for the elimination scenario, *Gap DFLE obs* gap in disability-free life expectancy between low and high educated, as observed in the data, *Gap DFLE UL* gap in disability-free life expectancy between low and high educated for the upward levelling scenario, *Gap DFLE elim* gap in disability-free life expectancy between low and high educated for the elimination scenario.

The numbers in the columns marked *change* are calculated as followed:

*Change [1] =*  *Gap TLE UL* – *Gap TLE obs, change [2] =*  *Gap TLE elim* – *Gap TLE obs, change [3] =*  *Gap DFLE UL – Gap DFLE obs,*

*change [4] =*  *Gap DFLE elim – Gap DFLE obs*

**Table A8** Comparison of total years lived with disease attributable to low fruit and vegetable intake in Global Burden of Disease study to unhealthy life years derived from the estimations based on the European Social Survey Round 7 for males and females, aged 35-79 years, in 10 European Countries.

|  |  | Global Burden of Disease study | | | ULY |  |
| --- | --- | --- | --- | --- | --- | --- |
| Country | Sex | Fruit | Vegetables | Combined | Combined | Ratio ESS:GBD |
|  |  | *% of total YLD* | *% of total YLD* | *% of total YLD* | *% of total ULY* |  |
| Finland | *Males* | 0.011 | 0.004 | 0.015 | 0.068 | 4.6 |
|  | *Females* | 0.006 | 0.003 | 0.009 | 0.047 | 5.0 |
| Denmark | *Males* | 0.010 | 0.004 | 0.014 | 0.081 | 5.7 |
|  | *Females* | 0.007 | 0.002 | 0.009 | 0.046 | 5.1 |
| UK | *Males* | 0.010 | 0.006 | 0.015 | 0.062 | 4.0 |
|  | *Females* | 0.006 | 0.004 | 0.010 | 0.046 | 4.6 |
| Belgium | *Males* | 0.011 | 0.004 | 0.015 | 0.061 | 4.2 |
|  | *Females* | 0.007 | 0.002 | 0.010 | 0.053 | 5.3 |
| Austria | *Males* | 0.009 | 0.004 | 0.013 | 0.094 | 7.4 |
|  | *Females* | 0.005 | 0.003 | 0.008 | 0.067 | 8.4 |
| Switzerland | *Males* | 0.008 | 0.003 | 0.011 | 0.060 | 5.4 |
|  | *Females* | 0.005 | 0.002 | 0.007 | 0.036 | 5.1 |
| Spain | *Males* | 0.010 | 0.003 | 0.013 | 0.073 | 5.5 |
|  | *Females* | 0.006 | 0.002 | 0.008 | 0.055 | 6.5 |
| Poland | *Males* | 0.015 | 0.007 | 0.022 | 0.063 | 2.9 |
|  | *Females* | 0.009 | 0.004 | 0.013 | 0.044 | 3.3 |
| Lithuania | *Males* | 0.017 | 0.008 | 0.025 | 0.077 | 3.0 |
|  | *Females* | 0.013 | 0.006 | 0.019 | 0.077 | 4.1 |
| Estonia | *Males* | 0.015 | 0.007 | 0.022 | 0.069 | 3.1 |
|  | *Females* | 0.012 | 0.005 | 0.017 | 0.062 | 3.7 |

*ULY* unhealthy life years, *ESS* European Social Survey, *GBD* Global Burden of Disease study, *YLD* years lived with disability.

Life expectancy with disability or unhealthy life years (ULY) were calculated by subtracting the estimations for disability-free life expectancy and for total life expectancy as calculated previously.

**Table A9** Educational differences in total life expectancy and disability-free life expectancy with 95% confidence interval by scenario for males and females, aged 35-79 years, in 10 European countries, 2006-2015 - extended version of Table 3

| MALES | OBSERVED |  |  | UPWARD LEVELLING |  |  |  |  | ELIMINATION |  |  |  |  |  |
| --- | --- | --- | --- | --- | --- | --- | --- | --- | --- | --- | --- | --- | --- | --- |
|  | Low  Educated | High  Educated | Gap  low vs high | Low  Educated | High Educated | Gap  low vs high | Change gap | Change gap in % | Low Educated | High Educated | Gap  low vs high | Change gap | Change gap in % | % of total change gap by upward levelling |
|  | **[A]** | **[B]** | **[C]** | **[D]** | **[E]** | **[F]** | **[G]** | **[H]** | **[I]** | **[J]** | **[K]** | **[L]** | **[N]** | **[M]** |
| Finland |  |  |  |  |  |  |  |  |  |  |  |  |  |  |
| *TLE* | 37.42  (37.30– 37.54) | 41.54  (41.48 – 41.61) | 4.11  (3.98 – 4.26) | 37.58  (37.39 – 37.76) | 41.54  (41.48 – 41.61) | 3.96  (3.78 – 4.16) | 0.15  (0.01 – 0.30) | 3.73 | 38.02  (37.86 – 38.16) | 41.75  (41.67 – 41.83) | 3.73  (3.57 – 3.91) | 0.38  (0.26 – 0.51) | 9.28 | *40.3* |
| *DFLE* | 23.65  (23.70 – 24.63) | 31.78  (30.92 – 32.61) | 8.12  (6.85 – 9.45) | 24.08  (23.06 – 25.15) | 31.78  (30.92 – 32.61) | 7.70  (6.33 – 9.04) | 0.42  (0.02 – 0.83) | 5.20 | 25.27  (24.38 – 26.27) | 32.59  (31.79 – 33.42) | 7.32  (6.06 – 8.57) | 0.80  (0.43 – 1.17) | 9.89 | *52.6* |
| Denmark |  |  |  |  |  |  |  |  |  |  |  |  |  |  |
| *TLE* | 37.49  (37.39 – 37.59) | 41.70  (41.64 – 41.76) | 4.21  (4.10 – 4.33) | 37.76  (37.62 – 37.90) | 41.70  (41.64 – 41.76) | 3.94  (3.80 – 4.10) | 0.27  (0.16 – 0.37) | 6.32 | 38.17  (38.06 – 38.27) | 41.90  (41.83 – 41.97) | 3.73  (3.61 – 3.80) | 0.48  (0.41 – 0.55) | 11.49 | *55.1* |
| *DFLE* | 25.93  (24.58 – 27.29) | 32.40  (31.27 – 33.51) | 6.47  (4.63 – 8.25) | 26.61  (25.29 – 27.96) | 32.40  (31.27 – 33.51) | 5.79  (4.00– 7.54) | 0.67  (0.41 – 0.95) | 10.46 | 27.65  (26.40 – 28.91) | 33.18  (32.13 – 34.23) | 5.53  (3.85 – 7.17) | 0.94  (0.68 – 1.18) | 14.60 | *71.6* |
| United Kingdom |  |  |  |  |  |  |  |  |  |  |  |  |  |  |
| *TLE* | 39.40  (39.11 – 39.71) | 42.09  (41.85 – 42.34) | 2.69  (2.31 – 3.07) | 39.59  (39.29 – 39.89) | 42.09  (41.85 – 42.34) | 2.50  (2.13 – 2.88) | 0.19  (0.11 – 0.27) | 7.27 | 39.80  (39.52 – 40.09) | 42.21  (41.98 – 42.45) | 2.41  (2.05 – 2.76) | 0.29  (0.22 – 0.35) | 10.84 | *67.1* |
| *DFLE* | 26.35  (25.64 – 27.04) | 34.60  (33.96 – 35.18) | 8.26  (7.32 – 9.12) | 26.98  (26.25 – 27.69) | 34.60  (33.96 – 35.18) | 7.62  (6.64 – 8.55) | 0.64  (0.37 – 0.88) | 7.87 | 27.68  (27.00 – 28.33) | 35.01  (34.37 – 35.59) | 7.33  (6.41 – 8.21) | 0.93  (0.72 – 1.13) | 11.44 | *68.8* |
| Belgium |  |  |  |  |  |  |  |  |  |  |  |  |  |  |
| *TLE* | 38.47  (38.42 – 38.53) | 41.16  (41.11 – 41.21) | 2.69  (2.62 – 2.76) | 38.64  (38.53 – 38.74) | 41.16  (41.11 – 41.21) | 2.52  (2.41 – 2.64) | 0.16  (0.07 – 0.25) | 6.39 | 38.93  (38.85 – 39.01) | 41.35  (41.27 – 41.40) | 2.41  (2.31 – 2.50) | 0.28  (0.21 – 0.35) | 10.76 | *59.4* |
| *DFLE* | 24.86  (24.01 – 25.82) | 34.68  (33.97 – 35.32) | 9.81  (8.60 – 10.85) | 25.37  (24.53 – 26.29) | 34.68  (33.97 – 35.32) | 9.31  (8.06 – 10.36) | 0.50  (0.21 – 0.77) | 5.39 | 26.26  (25.49 – 27.11) | 35.13  (34.45 – 35.76) | 8.87  (7.69 – 9.87) | 0.95  (0.71 – 1.17) | 10.05 | *53.7* |
| Austria |  |  |  |  |  |  |  |  |  |  |  |  |  |  |
| *TLE* | 38.27  (38.13 – 38.41) | 41.77  (41.67 – 41.86) | 3.51  (3.34 – 3.67) | 38.23  (38.05 – 38.41) | 41.77  (41.67 – 41.86) | 3.54  (3.35 – 3.74) | -0.03  (-0.14 – 0.07) | -0.91 | 38.89  (38.76 – 39.03) | 42.10  (42.01 – 42.20) | 3.21  (3.06 – 3.38) | 0.29  (0.23 – 0.36) | 8.39 | *-10.9* |
| *DFLE* | 19.45  (18.11 – 20.79) | 30.95  (30.08 – 31.76) | 11.50  (9.95 – 13.10) | 19.33  (17.94 – 20.68) | 30.95  (30.08 – 31.76) | 11.62  (9.99 – 13.23) | -0.12  (-0.50 – 0.28) | -1.04 | 21.80  (20.58 – 23.04) | 32.40  (31.60 – 33.21) | 10.60  (9.14 – 12.03) | 0.90  (0.60 – 1.19) | 7.90 | *-13.2* |
| Switzerland |  |  |  |  |  |  |  |  |  |  |  |  |  |  |
| *TLE* | 39.13  (38.99 – 39.26) | 42.29  (42.23 – 42.35) | 3.15  (3.01 – 3.32) | 39.30  (39.15 – 39.46) | 42.29  (42.23 – 42.35) | 2.98  (2.81 – 3.16) | 0.17  (0.09 – 0.26) | 5.50 | 39.55  (39.42 – 39.69) | 42.41  (42.34 – 42.34) | 2.85  (2.71 – 3.01) | 0.30  (0.24 – 0.36) | 9.66 | *57.0* |
| *DFLE* | 25.78  (23.79 – 28.01) | 34.18  (33.34 – 34.96) | 8.41  (6.18 – 10.59) | 26.33  (24.36 – 28.48) | 34.18  (33.34 – 34.96) | 7.86  (5.63 – 9.99) | 0.55  (0.28 – 0.86) | 6.70 | 27.15  (25.32 – 29.19) | 34.68  (33.86 – 35.43) | 7.53  (5.43 – 9.56) | 0.88  (0.62 – 1.16) | 10.66 | *62.8* |
| Spain |  |  |  |  |  |  |  |  |  |  |  |  |  |  |
| *TLE* | 39.24  (39.22 – 39.26) | 41.32  (41.28 – 41.35) | 2.08  (2.04 – 2.12) | 39.32  (39.23 – 39.40) | 41.32  (41.28 – 41.35) | 1.99  (1.91 – 2.09) | 0.08  (0.00 – 0.16) | 4.06 | 39.73  (39.69 – 39.78) | 41.58  (41.52 – 41.64) | 1.85  (1.78 – 1.92) | 0.23  (0.17 – 0.29) | 11.27 | *36.1* |
| *DFLE* | 28.21  (27.81 – 28.63) | 34.18  (33.55 – 34.75) | 5.97  (5.20 – 6.67) | 28.44  (27.96 – 28.90) | 34.18  (33.55 – 34.75) | 5.73  (4.98 – 6.47) | 0.23  (0.01 – 0.44) | 4.02 | 29.57  (29.16 – 29.96) | 34.92  (34.35 – 35.45) | 5.36  (4.66 – 6.02) | 0.61  (0.42 – 0.78) | 10.55 | *38.1* |
| Poland |  |  |  |  |  |  |  |  |  |  |  |  |  |  |
| *TLE* | 34.17  (34.14 – 34.19) | 40.66  (40.62 – 40.70) | 6.49  (6.45 – 6.54) | 34.22  (34.07 – 34.40) | 40.66  (40.62 – 40.70) | 6.44  (6.25 – 6.60) | 0.05  (-0.10 – 0.23) | 0.83 | 34.90  (34.82 – 34.97) | 40.98  (40.89 – 41.05) | 6.08  (5.97 – 6.18) | 0.41  (0.32 – 0.51) | 6.33 | *13.0* |
| *DFLE* | 22.07  (21.30 – 22.88) | 32.64  (31.81 – 33.49) | 10.57  (9.46 – 11.66) | 22.18  (21.35 – 23.04) | 32.64  (31.81 – 33.49) | 10.46  (9.28 – 11.59) | 0.11  (-0.20 – 0.47) | 1.03 | 23.55  (22.81 -24.29) | 33.51  (32.67 –34.33) | 9.96  (8.88 – 10.97) | 0.61  (0.37 – 0.85) | 5.68 | *38.1* |
| Lithuania |  |  |  |  |  |  |  |  |  |  |  |  |  |  |
| *TLE* | 31.25  (31.02 – 31.49) | 39.43  (39.29 – 39.58) | 8.19  (7.91 – 8.47) | 31.87  (31.58 – 32.09) | 39.43  (39.29 – 39.58) | 7.56  (7.21 – 7.89) | 0.63  (0.43 – 0.83) | 7.66 | 32.66  (32.42 – 32.90) | 39.85  (39.69 – 40.01) | 7.18  (6.90 – 7.48) | 1.01  (0.87 – 1.15) | 12.28 | *62.4* |
| *DFLE* | 19.19  (17.37 – 21.04) | 32.54  (31.60 – 33.54) | 13.36  (11.23 – 15.52) | 20.32  (18.58 – 22.09) | 32.54  (31.60 – 33.54) | 12.23  (10.08 – 14.22) | 1.13  (0.75 – 1.50) | 8.46 | 21.75  (20.11 – 23.34) | 33.44  (32.53 – 34.38) | 11.69  (9.72 – 13.57) | 1.66  (1.31 – 2.04) | 12.46 | *67.9* |
| Estonia |  |  |  |  |  |  |  |  |  |  |  |  |  |  |
| *TLE* | 32.84  (32.54 – 33.13) | 40.13  (39.93 – 40.33) | 7.29  (6.93 – 7.65) | 33.06  (32.70 – 33.42) | 40.13  (39.93 – 40.33) | 7.07  (6.67 – 7.48) | 0.22  (0.02 – 0.41) | 2.97 | 33.76  (33.44 – 34.08) | 40.45  (40.26 – 40.65) | 6.70  (6.32 – 7.07) | 0.59  (0.44 – 0.74) | 8.15 | *36.4* |
| *DFLE* | 16.65  (15.35 – 17.86) | 27.51  (26.50 – 28.53) | 10.86  (9.26 – 12.47) | 17.14  (15.86 – 18.35) | 27.51  (26.50 – 28.53) | 10.38  (8.68 – 12.01) | 0.49  (0.04 – 0.93) | 4.51 | 18.71  (17.47 – 19.82) | 28.62  (27.67 – 29.59) | 9.91  (8.36 – 11.44) | 0.95  (0.52 – 1.36) | 8.62 | *52.3* |
| All Countries |  |  |  |  |  |  |  |  |  |  |  |  |  |  |
| *TLE* | 37.22 | 41.50 | 4.29 | 37.41 | 41.50 | 4.09 | 0.19 |  | 37.82 | 41.70 | 3.88 | 0.41 |  |  |
| *DFLE* | 24.82 | 32.76 | 7.94 | 25.30 | 32.76 | 7.46 | 0.48 |  | 26.31 | 33.43 | 7.11 | 0.83 |  |  |

| FEMALES | OBSERVED |  |  | UPWARD LEVELLING |  |  |  |  | ELIMINATION |  |  |  |  |  |
| --- | --- | --- | --- | --- | --- | --- | --- | --- | --- | --- | --- | --- | --- | --- |
|  | Low  Educated | High  Educated | Gap  low vs high | Low  Educated | High Educated | Gap  low vs high | Change gap | Change gap in % | Low Educated | High Educated | Gap  low vs high | Change gap | Change gap in % | % of total change gap by upward levelling |
|  | [A] | [B] | [C] | [D] | [E] | [F] | [G] | [H] | [I] | [J] | [K] | [L] | [N] | [M] |
| Finland |  |  |  |  |  |  |  |  |  |  |  |  |  |  |
| *TLE* | 40.63  (40.50 – 40.74) | 43.03  (42.98 – 43.08) | 2.40  (2.28 – 2.54) | 40.82  (40.68 – 40.96) | 43.03  (42.98 – 43.08) | 2.21  (2.06 – 2.36) | 0.20  (0.11 – 0.28) | 8.00 | 40.94  (40.82 – 41.07) | 43.08  (43.03 – 43.13) | 2.14  (2.00 – 2.29) | 0.26  (0.19 – 0.33) | 10.66 | *75.1* |
| *DFLE* | 24.33  (23.10 – 25.50) | 29.26  (28.32 – 30.07) | 4.93  (3.43 – 6.42) | 25.27  (24.07 – 26.48) | 29.26  (28.32 – 30.07) | 3.99  (2.44 – 5.50) | 0.94  (0.53 – 1.32) | 18.07 | 25.84  (24.67 – 26.98) | 29.74  (28.85 – 30.52) | 3.90  (2.38 – 5.35) | 1.03  (0.65 – 1.40) | 20.02 | *90.2* |
| Denmark |  |  |  |  |  |  |  |  |  |  |  |  |  |  |
| *TLE* | 39.95  (39.87 – 40.04) | 42.60  (42.55 – 42.65) | 2.65  (2.54 – 2.74) | 40.09  (39.98 – 40.20) | 42.60  (42.55 – 42.65) | 2.51  (2.39 – 2.64) | 0.14  (0.07 – 0.20) | 5.19 | 40.22  (40.12 – 40.31) | 42.66  (42.61 – 42.72) | 2.45  (2.33 – 2.56) | 0.20  (0.14 – 0.25) | 7.58 | *68.5* |
| *DFLE* | 26.20  (24.98 – 27.48) | 30.64  (29.53 – 31.78) | 4.44  (2.67 – 6.19) | 26.71  (25.49 – 27.94) | 30.64  (29.53 – 31.78) | 3.93  (2.12 – 5.69) | 0.51  (0.25 – 0.74) | 11.63 | 27.20  (26.06 – 28.39) | 31.05  (29.96 – 32.17) | 3.85  (2.09 – 5.58) | 0.59  (0.34 – 0.80) | 13.47 | *86.4* |
| United Kingdom |  |  |  |  |  |  |  |  |  |  |  |  |  |  |
| *TLE* | 41.04  (40.80 – 41.28) | 42.71  (42.46 – 42.96) | 1.66  (1.33 – 2.00) | 41.20  (40.97 – 41.44) | 42.71  (42.46 – 42.96) | 1.50  (1.19 – 1.83) | 0.16  (0.12 – 0.21) | 9.88 | 41.30  (41.08 – 41.53) | 42.76  (42.52 – 43.01) | 1.46  (1.16 – 1.79) | 0.20  (0.16 – 0.25) | 12.21 | *80.9* |
| *DFLE* | 26.78  (26.14 – 27.49) | 33.52  (33.82 – 34.20) | 6.73  (5.76 – 7.69) | 27.53  (26.88– 28.24) | 33.52  (32.82 – 34.20) | 5.98  (4.98 – 6.93) | 0.75  (0.55 – 0.96) | 7.87 | 27.99  (27.38 – 28.65) | 33.80  (33.10 – 34.49) | 5.81  (4.84 – 6.73) | 0.92  (0.74 – 1.11) | 11.44 | *81.7* |
| Belgium |  |  |  |  |  |  |  |  |  |  |  |  |  |  |
| *TLE* | 41.16  (41.11 – 41.21) | 42.56  (42.51 – 42.61) | 1.40  (1.33 – 1.46) | 41.23  (41.15 – 41.30) | 42.56  (42.51 – 42.61) | 1.33  (1.25 – 1.43) | 0.07  (0.01 – 0.12) | 4.96 | 41.38  (41.32 – 41.44) | 42.66  (42.61 – 42.71) | 1.28  (1.20 – 1.36) | 0.12  (0.08 – 0.16) | 9.10 | *54.5* |
| *DFLE* | 24.49  (23.61 – 25.39) | 33.59  (32.69 – 34.41) | 9.11  (7.83 – 10.28) | 24.84  (23.95 – 25.78) | 33.59  (32.69 – 34.41) | 8.76  (7.46 – 9.96) | 0.35  (0.06 – 0.63) | 4.07 | 25.66  (24.84 – 26.49) | 34.02  (33.16 – 34.84) | 8.37  (7.13 – 9.51) | 0.74  (0.50 – 0.96) | 8.45 | *48.2* |
| Austria |  |  |  |  |  |  |  |  |  |  |  |  |  |  |
| *TLE* | 41.47  (41.39 – 41.55) | 42.82  (42.72 – 42.93) | 1.35  (1.21 – 1.49) | 41.58  (41.48 – 41.68) | 42.82  (42.72 – 42.93) | 1.24  (1.09 – 1.39) | 0.11  (0.05 – 0.17) | 8.24 | 41.77  (41.69 – 41.85) | 42.94  (42.83 – 43.05) | 1.17  (1.04 – 1.31) | 0.18  (0.14 – 0.22) | 13.40 | *61.5* |
| *DFLE* | 22.23  (21.30 – 23.21) | 30.63  (29.55 – 31.74) | 8.41  (6.88 – 9.94) | 22.92  (21.92 – 23.94) | 30.63  (29.55 – 31.74) | 7.71  (6.17 – 9.29) | 0.69  (0.31 – 1.07) | 8.45 | 24.11  (23.24 – 25.06) | 31.42  (30.32 – 32.53) | 7.31  (5.86 – 8.78) | 1.10  (0.81 – 1.37) | 13.47 | *62.8* |
| Switzerland |  |  |  |  |  |  |  |  |  |  |  |  |  |  |
| *TLE* | 41.94  (41.86 – 42.02) | 43.11  (43.04 – 43.19) | 1.18  (1.06 – 1.28) | 41.93  (41.84 – 42.03) | 43.11  (43.04 – 43.19) | 1.18  (1.05 – 1.30) | 0.00  (-0.05 – 0.04) | -0.22 | 42.06  (41.97 – 42.13) | 43.19  (43.11 – 43.27) | 1.14  (1.01 – 1.24) | 0.04  (0.01 – 0.08) | 3.56 | *-6.2* |
| *DFLE* | 27.88  (26.55 – 29.20) | 31.57  (30.31 – 32.74) | 3.69  (1.74 – 5.63) | 27.87  (26.43 – 29.21) | 31.57  (30.31 – 32.74) | 3.71  (1.78 – 5.67) | -0.01  (-0.31 – 0.25) | -0.39 | 28.54  (27.21 – 29.81) | 32.12  (30.94 – 33.25) | 3.58  (1.73 – 5.43) | 0.12  (0.13 – 0.34) | 3.11 | *-12.7* |
| Spain |  |  |  |  |  |  |  |  |  |  |  |  |  |  |
| *TLE* | 42.38  (42.37 – 42.40) | 42.94  (42.91 – 42.98) | 0.56  (0.52 – 0.60) | 42.43  (42.39 – 42.47) | 42.94  (42.91 – 42.98) | 0.51  (0.46 – 0.57) | 0.05  (0.01 – 0.08) | 8.38 | 42.55  (42.53 – 42.58) | 43.04  (43.00 – 43.08) | 0.49  (0.44 – 0.54) | 0.07  (0.04 – 0.10) | 13.16 | *63.6* |
| *DFLE* | 27.91  (27.47 – 28.34) | 34.79  (34.09 – 35.44) | 6.89  (6.05 – 7.71) | 28.21  (27.72 – 28.68) | 34.79  (34.09 – 35.44) | 6.59  (5.75 – 7.44) | 0.30  (0.04 – 0.53) | 4.38 | 29.00  (28.59 – 29.44) | 35.27  (34.59 – 35.91) | 6.27  (5.48 – 7.08) | 0.62  (0.43 – 0.79) | 8.99 | *48.7* |
| Poland |  |  |  |  |  |  |  |  |  |  |  |  |  |  |
| *TLE* | 39.80  (39.78 – 39.83) | 42.52  (42.49 – 42.54) | 2.71  (2.68 – 2.75) | 39.99  (39.91 – 40.06) | 42.52  (42.49 – 42.54) | 2.53  (2.45 – 2.61) | 0.18  (0.10 – 0.25) | 6.64 | 40.14  (40.09 – 40.18) | 42.59  (42.55 – 42.64) | 2.46  (2.40 – 2.52) | 0.25  (0.20 – 0.30) | 9.20 | *72.1* |
| *DFLE* | 24.75  (23.89 – 25.55) | 32.31  (31.50 – 33.08) | 7.56  (6.45 – 8.73) | 25.46  (24.59 – 26.24) | 32.31  (31.50 – 33.08) | 6.85  (5.76 – 8.01) | 0.71  (0.41 – 0.97) | 9.13 | 26.04  (25.23 – 26.81) | 32.71  (31.94 – 33.47) | 6.67  (5.60– 7.78) | 0.89  (0.65 – 1.10) | 11.55 | *79.1* |
| Lithuania |  |  |  |  |  |  |  |  |  |  |  |  |  |  |
| *TLE* | 37.84  (37.58 – 38.11) | 42.32  (42.22 – 42.40) | 4.47  (4.20 – 4.75) | 38.26  (37.98 – 38.55) | 42.32  (42.22 – 42.40) | 4.06  (3.75 – 4.34) | 0.42  (0.29 – 0.53) | 9.25 | 38.58  (38.33 – 38.85) | 42.46  (42.37 – 42.55) | 3.88  (3.60 – 4.13) | 0.60  (0.50- 0.69) | 13.33 | *69.4* |
| *DFLE* | 21.15  (18.78 – 23.56) | 34.58  (33.64 – 35.49) | 13.43  (10.82 – 16.10) | 22.48  (20.17 – 24.72) | 34.58  (33.64 – 35.49) | 12.10  (9.51 – 14.70) | 1.33  (0.92 – 1.73) | 9.87 | 23.53  (21.42 – 25.64) | 35.12  (34.22 – 35.98) | 11.59  (9.14 – 14.03) | 1.84  (1.44 – 2.24) | 13.66 | *72.2* |
| Estonia |  |  |  |  |  |  |  |  |  |  |  |  |  |  |
| *TLE* | 38.69  (38.31 – 39.04) | 42.59  (42.48 – 42.71) | 3.90  (3.52 – 4.27) | 38.92  (38.55 – 39.30) | 42.59  (42.48 – 42.71) | 3.67  (3.27 – 4.05) | 0.23  (0.10 – 0.36) | 7.01 | 39.21  (38.85 – 39.58) | 42.73  (42.61 – 42.84) | 3.51  (3.13 – 3.87) | 0.39  (0.28 – 0.50) | 10.81 | *64.8* |
| *DFLE* | 18.04  (16.53 – 19.58) | 29.56  (28.74- 30.38) | 11.52  (9.88 – 13.25) | 18.87  (17.32 – 20.40) | 29.56  (28.74 – 30.38) | 10.69  (8.96 – 12.45) | 0.83  (0.32 – 1.33) | 9.74 | 20.18  (18.78 – 21.60) | 30.48  (29.70 – 31.22) | 10.30  (8.65 – 11.95) | 1.22  (0.77 – 1.69) | 13.03 | *74.8* |
| All Countries |  |  |  |  |  |  |  |  |  |  |  |  |  |  |
| *TLE* | 41.16 | 42.70 | 1.54 | 41.29 | 42.70 | 1.42 | 0.13 |  | 41.41 | 42.78 | 1.37 | 0.17 |  |  |
| *DFLE* | 25.72 | 31.66 | 5.94 | 26.35 | 31.66 | 5.32 | 0.63 |  | 26.96 | 32.10 | 5.14 | 0.80 |  |  |

*TLE* total life expectancy, *DFLE* disability-free life expectancy. Calculations: [C] = [B] – [A]. [F] = [E] – [D]. [K] = [J] – [I].

[G] = [C] – [F]. [L] = [C] –[K]. [H] = [G] / [C]. [N] = [L] / [C]. [M] = [H] / [N]. Since the estimates presented here were rounded after finishing all calculations, reproducing the estimates by hand will yield slightly different results.

References

Wang X, Ouyang YY, Liu J, Zhu MM, Zhao G, Bao W, Hu FB (2014) Fruit and vegetable consumption and mortality from all causes, cardiovascular disease, and cancer: systematic review and dose-response meta-analysis of prospective cohort studies. Bmj-British Medical Journal 349. doi: Artn G4490

10.1136/Bmj.G4490
